# Exploring digenic inheritance in arrhythmogenic cardiomyopathy

**DOI:** 10.1186/s12881-017-0503-7

**Published:** 2017-12-08

**Authors:** Eva König, Claudia Béu Volpato, Benedetta Maria Motta, Hagen Blankenburg, Anne Picard, Peter Pramstaller, Michela Casella, Werner Rauhe, Giulio Pompilio, Viviana Meraviglia, Francisco S. Domingues, Elena Sommariva, Alessandra Rossini

**Affiliations:** 1 Institute for Biomedicine, Eurac Research, Affiliated Institute of the University of Lübeck, Bolzano, Italy; 2 Heart Rhythm Center, Centro Cardiologico Monzino IRCCS, Via Parea 4, 20138 Milan, Italy; 3 General Hospital Bolzano/Bozen, Bolzano, Italy; 4 Vascular Biology and Regenerative Medicine Unit, Centro Cardiologico Monzino IRCCS, Via Parea 4, 20138 Milan, Italy

**Keywords:** Digenic inheritance, Arrhythmogenic cardiomyopathy, Exome sequencing, ACM, PKP2

## Abstract

**Background:**

Arrhythmogenic cardiomyopathy (ACM) is an inherited genetic disorder, characterized by the substitution of heart muscle with fibro-fatty tissue and severe ventricular arrhythmias, often leading to heart failure and sudden cardiac death. ACM is considered a monogenic disorder, but the low penetrance of mutations identified in patients suggests the involvement of additional genetic or environmental factors.

**Methods:**

We used whole exome sequencing to investigate digenic inheritance in two ACM families where previous diagnostic tests have revealed a *PKP2* mutation in all affected and some healthy individuals. In family members with *PKP2* mutations we determined all genes that harbor variants in affected but not in healthy carriers or vice versa. We computationally prioritized the most likely candidates, focusing on known ACM genes and genes related to *PKP2* through protein interactions, functional relationships, or shared biological processes.

**Results:**

We identified four candidate genes in family 1, namely *DAG1*, *DAB2IP*, *CTBP2* and *TCF25,* and eleven candidate genes in family 2. The most promising gene in the second family is *TTN*, a gene previously associated with ACM, in which the affected individual harbors two rare deleterious-predicted missense variants, one of which is located in the protein’s only serine kinase domain.

**Conclusions:**

In this study we report genes that might act as digenic players in ACM pathogenesis, on the basis of co-segregation with *PKP2* mutations. Validation in larger cohorts is still required to prove the utility of this model.

**Electronic supplementary material:**

The online version of this article (10.1186/s12881-017-0503-7) contains supplementary material, which is available to authorized users.

## Background

Arrhythmogenic cardiomyopathy (ACM) is a genetic disorder in which the ventricular myocardium is progressively replaced by fibro-fatty tissue. Since this occurs predominantly in the right ventricle, the disease is also known as arrhythmogenic right ventricular cardiomyopathy (ARVC). ACM is associated with progressive heart failure and severe ventricular arrhythmias, often leading to sudden death, especially in young people and athletes [1]. About half of the affected individuals harbor mutations in one of the five genes of the cardiac desmosome (*PKP2*, *JUP*, *DSP*, *DSG2*, *DSC2*), of which mutations in *PKP2* are most common. Desmosomes are intercellular junctions that confer strong cell–cell adhesion and provide a mechanical connection between cardiomyocytes. Therefore, desmosomal defects can have deleterious effects on tissue integrity. In addition, desmosomal proteins play an important role in signaling and regulation of cell proliferation and differentiation [2]. In ACM, pathogenic mechanisms include suppression of Wnt signaling and activation of the Hippo pathway [3] leading to adipogenesis. Beside desmosomal genes, mutations in eight additional genes (*DES*, *PLN*, *TGFB3*, *CTNNA3*, *LMNA*, *TMEM43*, *RYR2*, and *TTN*) have been found to cause ACM [1]. Recently, two studies reported *FLNC* and *CDH2* as possible novel causative genes for ACM [4, 5]. In most patients, ACM is inherited in an autosomal dominant mode with reduced penetrance (not all individuals with a causal mutation develop ACM) and variable expressivity (the severity and the nature of the symptoms may vary between affected individuals, even if they have the same causal mutation).

Autosomal dominant mutations have only been identified in up to 60% of all ACM patients [1] suggesting the existence of unknown mechanisms such as higher genetic heterogeneity, modifier genes, or cross talk between genetic background and environmental factors [6]. In fact, three loci have been mapped in ACM linkage studies, for which the causal gene has not yet been identified: ARVD3 (OMIM %602,086) at 14q12–32.3 [7], ARVD4 (OMIM %602,087) at 2q32.1–32.3 [8], and ARVD6 (OMIM %604,401) at 10p14-p12 [9]. In addition, the frequency of variants associated with ACM has been found to be much higher than expected given the phenotype prevalence in the general population, suggesting that a high number of these variants are not monogenic causes of ACM [10]. In fact, recent reports have suggested digenic inheritance as an alternative disease mechanism of ACM [11–14]. In digenic inheritance the presence of two variants in two different genes is required for the manifestation of a clinical phenotype; in the absence of one of these variants, the other variant might be benign. For example, Xu et al. screened 198 ACM patients for variants in the desmosomal genes. Of the 38 patients in which *PKP2* variants were detected, additional variants in *PKP2* itself (compound heterozygosity) were identified in nine patients; variants in other desmosomal genes (digenic inheritance) were identified in 13 patients. Related family members harboring a variant in just one of these genes were unaffected by ACM. The authors concluded that the disease was caused by compound heterozygosity or digenic inheritance in these patients [12]. Rasmussen et al. investigated 12 families with variants in *DSG2*. In three of these families, additional variants were identified in the *DSP* gene in affected family members. Only individuals with both variants in *DSG2* and *DSP* were affected by ACM, leading the authors to conclude that low penetrance of desomosmal variants in ACM patients may also be explained by digenic inheritance [13]. Cooper et al. proposed that digenic inheritance may occur as a result of variants in two genes encoding different subunits of the same protein (complex); two proteins that interact functionally; are a receptor-ligand pair; are a target gene and transcription factor; or compromise the same regulatory, biosynthetic, or degradative pathway [11]. Digenic inheritance is distinct from modifier genes: in digenic inheritance, both variants individually usually do not lead to disease, whereas in modifier genes one pathogenic variant is enhanced by a putatively contributing variant of unknown significance [15]. Non-genetic factors known to influence ACM penetrance are age, male sex and intense physical activity [16, 17].

We performed whole exome sequencing on two families, in which diagnostic tests have identified a *PKP2* mutation in affected and healthy individuals. Assuming a digenic mode of inheritance, we determined all genes, where in addition to the observed *PKP2* variant a second causal variant was expected to be present in either the affected individuals or the *PKP2* carriers. Filtering and prioritizing these genes, we determined four candidate genes in the first, and eleven candidate genes for digenic inheritance in the second family.

## Methods

### Subjects

**Fig. 1:**
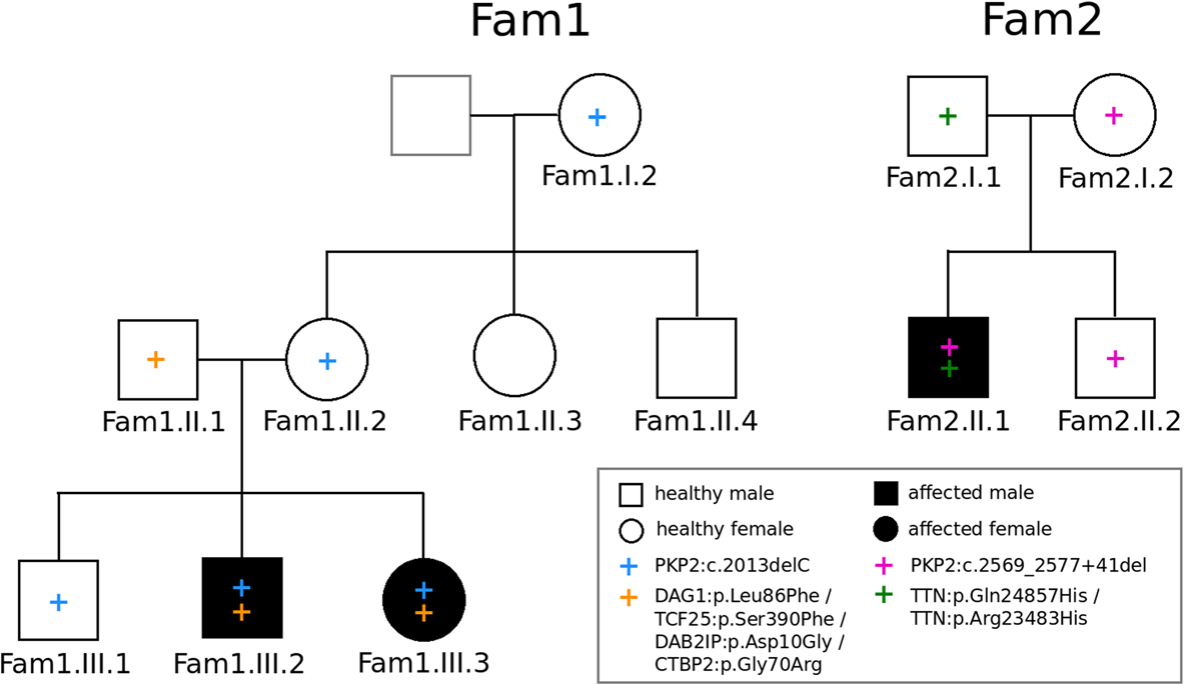
Pedigrees of the two families analyzed in this study. Only labeled individuals were sequenced. +: Heterozygous variant, filled black symbols: *affected* individuals; white symbols with +: *carrier* individuals; white empty symbols: *healthy* individuals. Left: Family 1 (Fam1); the blue + symbol indicates the presence of the *NM_004572.3(PKP2)*:c.2013delC variant, the orange + symbol indicates the presence of the four Fam1 variants *ENSP00000312435.2(DAG1)*:p.Leu86Phe, *ENSP00000263347.7(TCF25)*:p.Ser390Phe, *ENSP00000259371.2(DAB2IP)*:p.Asp10Gly, *ENSP00000357816.5(CTBP2)*:p.Gly70Arg. Right: Family 2 (Fam2); the pink + symbol indicates the presence of the *NG_009000.1(PKP2)*:c.2569_2577 + 41del variant, the green + symbol indicates the presence of the ENSP00000434586.1(*TTN*):p.Gln24857His and the ENSP00000434586.1(*TTN*):p.Arg23483His variants

In this study two Italian families comprising eight and four individuals, respectively, were investigated. Two individuals in the first and one individuals in the second family have been diagnosed with arrhythmogenic cardiomyopathy (ACM) according to the diagnostic task force criteria [18] (Table 1). Furthermore, previous clinically certified molecular tests on known ACM related genes have identified *PKP2* variants in all affected and some healthy family members (Fig. 1).

**Table 1:** Clinical characteristics of studied individuals^a^

Family	ID	Gender	Age	Physical exercise	Affected by ACM	Type of first symptom	Age at first symptom	Diagnosed ACM mutation	ICD	ACM Therapy	Dysfunction and structural alterations	Tissue characteristic of wall	Repolarization abnormalities	Depolarization or conduction abnormalities	Arrhythmias	Comorbidities
Fam1	Fam1.I.2	F	87	no	no	–	–	NM_004572.3(PKP2):c.2013delC	no	none	none	n.a.	none	none	none	hypertention
	Fam1.II.1	M	70	no	no	**–**	**–**	**–**	no	none	n.a.	n.a.	none	none	none	hyperlipidemia
	Fam1.II.2	F	67	no	no	–	–	NM_004572.3(PKP2):c.2013delC	no	none	none	n.a.	minor	none	none	hyperlipidemia
	Fam1.II.3	F	62	no	no	–	–	–	no	none	n.a.	n.a.	none	none	none	hyperlipidemia
	Fam1.II.4	M	68	no	no	–	–	–	no	none	n.a.	n.a.	none	none	none	myocardial infarction
	Fam1.III.1	M	39	yes	no	–	–	NM_004572.3(PKP2):c.2013delC	no	none	none	n.a.	minor	none	none	none
	Fam1.III.2	M	35	no	yes	VT	21	NM_004572.3(PKP2):c.2013delC	yes	Sotalol	major	n.a.	major	major	major	hyperlipidemia
	Fam1.III.3	F	31	yes	yes	Syncope	17	NM_004572.3(PKP2):c.2013delC	yes	Sotalol	major	n.a.	major	none	major	none
Fam2	Fam2.I.1	M	67	no	no	–	–	–	no	none	n.a.	n.a.	none	none	none	none
	Fam2.I.2	F	66	no	no	–	–	NG_009000.1(PKP2):c.2569_2577 + 41del	no	none	none	n.a.	minor	none	none	atrial fibrillation
	Fam2.II.1	M	34	yes	yes	Syncope	24	NG_009000.1(PKP2):c.2569_2577 + 41del	yes	Sotalol	minor	n.a.	major	major	major	none
	Fam2.II.2	M	41	yes	no	–	–	NG_009000.1(PKP2):c.2569_2577 + 41del	no	none	none	n.a.	none	none	none	none

^a^Individuals classified as they reach major or minor diagnostic criteria [18]

n.a.: not available; VT: Ventricular Tachycardia; ICD: implantable cardioverter defibrillator; Athletic lifestyle: defined as intense sportive activity more than 3 times a week

Family 1 (Fam1) consists of eight individuals in three generations of which two are affected by ACM (Fam1.III.2 and Fam1.III.3). The five individuals Fam1.I.2, Fam1.II.2, Fam1.III.1, Fam1.III.2, and Fam1.III.3 carry the heterozygous one base-pair deletion *NM_004572.3(PKP2)*:c.2013delC, *NP_004563.2(PKP2)*:p.Lys672ArgfsTer12, which results in a premature stop codon after a frameshift mutation.

Family 2 (Fam2) consists of two parents and their two sons, one of which (Fam2.II.1) is affected by ACM. The male patient Fam2.II.1, his brother Fam2.II.2 and their mother Fam2.I.2 carry the heterozygous nine base pair deletion *NG_009000.1(PKP2)*:c.2569_2577 + 41del, which crosses an exon/intron border.

In this study, ACM-diagnosed individuals are referred to as *affecte*d, healthy individuals with a *PKP2* mutation are called *carriers*, and healthy individuals without a *PKP2* mutation are called *healthy*.

Details on the diagnostic genetic tests are given in the Additional file 1.

### Data generation and computational processing

Samples were prepared following the Nextera® Rapid Capture Exome Enrichment kit protocol and were sequenced on two lanes of a HiSeq 2500 in paired end mode (2 × 100). Reads were aligned with BWA [19] and variants called with GATK [20], following the best practice recommendations. Variants were annotated with information from Ensembl [21], the ExAC project [22] (allele frequency (AF), variants with AF < 0.01 are called *rare*), PROVEAN deleteriousness prediction scores (variants with scores < −2.5 are called *deleterious*) [23] (for SNPs and indels), and LR.PF3 pathogenicity prediction scores [24] (for SNPs only). Copy number variations (CNVs) were called with XHMM [25]. MAESTROweb [26, 27] was used to predict the effect of variants on protein stability based on protein structure, where structure data were available. Sequence conservation was computed with ConSurf [28]. A detailed description is given in the Additional file 1.

### Family-based gene selection

To investigate whether individuals in the two families develop ACM if they carry the *PKP2* mutation and a variant that affects a second unknown gene, a set of putative causal genes was compiled in each family. All genes were determined that have a least one variant that meets the following three criteria: (i) The variant has a consequence, that is classified as either “high” (transcript ablation, splice acceptor variant, splice donor variant, stop gained, frameshift variant, stop lost, start lost, transcript amplification) or “moderate” (inframe insertion, inframe deletion, missense variant, protein altering variant) by Ensembl. (ii) The variant is either present in the family’s affected individuals and not in any family’s *PKP2* carrier individuals or it is present in the family’s *PKP2* carrier individuals and not in any family’s affected individuals in either a dominant or a recessive mode. (iii) The variant has a coverage of at least 10X in all affected and carrier individuals.

In addition to the Fam1 and Fam2 family members, an unrelated female ACM affected individual, her carrier sister, and their carrier aunt, all carrying a heterozygous *PKP2* exon 4 deletion, were used to exclude variants as described in (ii) (see Additional file 1 for details). Variants were not filtered based on allele frequency or pathogenicity prediction. Furthermore, all genes were determined that harbored copy number variations (CNV; either a deletion or a duplication) in the affected and carrier individuals applying the same genotype selection criteria as for variants.

**Fig. 2:**
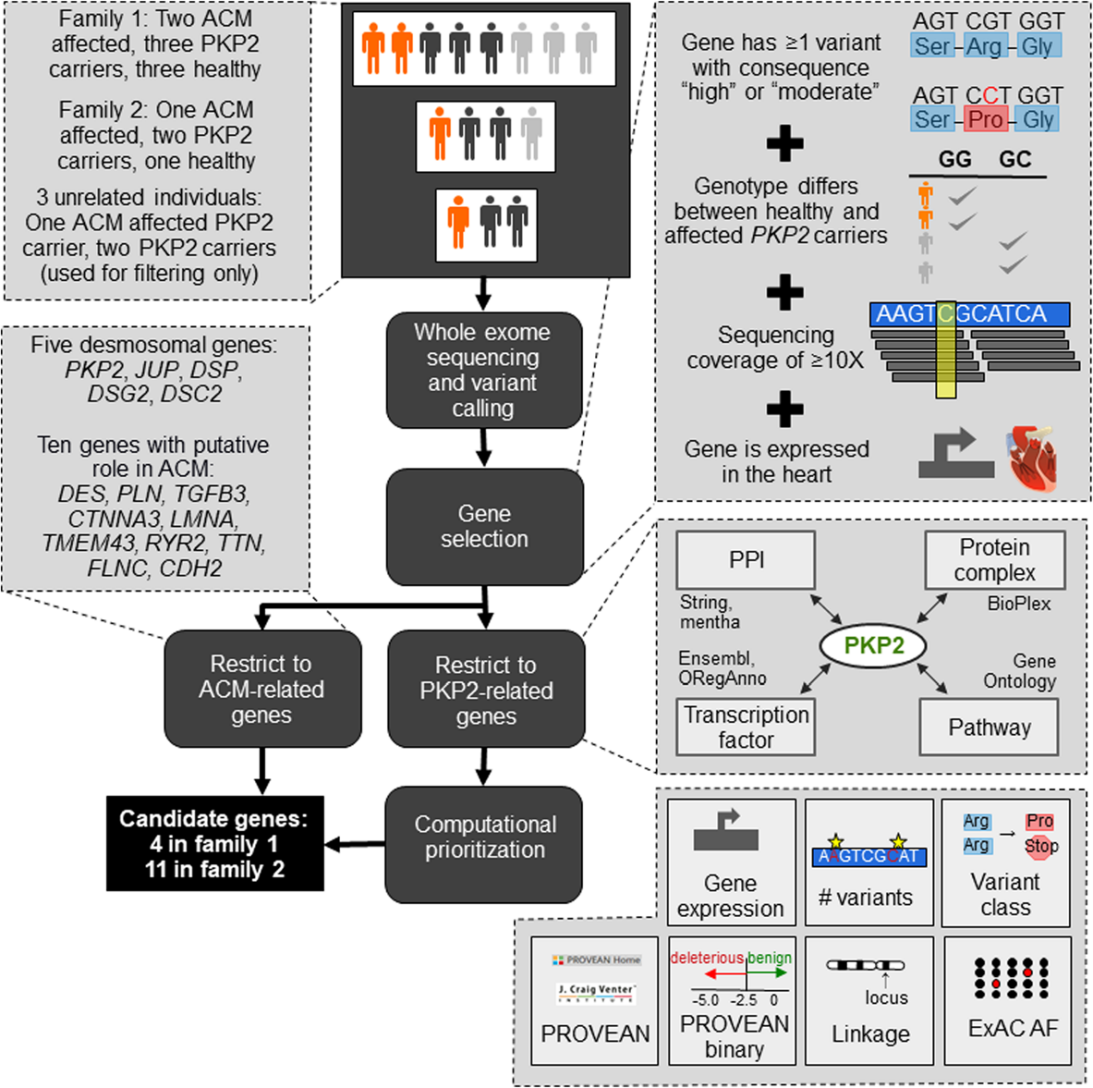
Methods summary. Whole exome sequencing and variant calling was performed for each individual in both families. For each family, genes are selected that have at least one variant with a high or moderate variant impact, that differ between healthy and affected *PKP2* carriers, that have a coverage of at least 10X, and that are expressed in the heart. These genes are filtered to include only genes related to ACM or *PKP2* and are computationally prioritized. Results are presented in Table 2

Each family’s gene set was filtered to only include genes expressed in the heart. The RNA gene dataset was downloaded from the ProteinAtlas [29] version 15, which contains gene expression levels of 45 cell lines and 32 tissues based on RNA-seq. A gene was considered *expressed in the heart*, if it had an expression level of at least 5 FPKM in the heart muscle in this dataset. We call the genes/variants determined by these filtering steps *Fam1 genes/variants* and *Fam2 genes/variants*. This gene selection is visualized in Fig. 2.

### ACM and *PKP2*-related genes

A set of 15 genes known to be involved in ACM was created by literature review [4, 5, 30]. In particular, the ACM gene set consists of the desmosomal genes *PKP2*, *JUP*, *DSP*, *DSG2*, and *DSC2* and the genes *DES*, *PLN*, *RYR2*, *TGFB3*, *TMEM43*, *TTN*, *CTNNA3*, *LMNA, FLNC,* and *CDH2*.

Since *PKP2* is relevant for the development of ACM in both families, we assumed that the second unknown gene is directly related to *PKP2* [11]*.* Following the probable mechanisms of digenic inheritance described by Cooper et al. [11], a set of *PKP2-*related genes was compiled based on the following five criteria. (i) *The two gene products form a protein complex.* Gene complex data were downloaded from the BioPlex database [31] version 4. Genes that interacted with *PKP2* with a confidence of at least 0.7 were selected. (ii) *The two gene products interact functionally. PKP2* interactors were downloaded from the STRING database [32] version 10 and the mentha database [33] version 2016–08-07. STRING interactions were restricted to those with a confidence of at least 0.7. The union of *PKP2* interactors from the two databases was selected. (iii) *The two gene products are transcription factor and target gene.* The Ensembl database [21] version 84 and ORegAnno database [34] (release 2015.12.22) were manually reviewed for transcription factors of *PKP2*. (iv) *The two gene products participate in the same pathway.* “Biological process” (BP) gene annotations from the Gene Ontology [35] (GO) were used as an approximation. All biological process annotations of *PKP2* and all their annotated proteins were queried from the GO database version 2016.7 using the Dintor GOAnnotator tool [36]. Processes were restricted to those where at least half of their annotated genes were expressed in the heart and all genes annotated to these processes were selected, creating a set of genes that share a biological function with *PKP2*. (v) *The two genes are paralogs*. Though not specifically listed as a mechanism of digenic inheritance, Cooper et al. discussed that paralogous genes might provide a level of redundancy by resuming gene function in case of a disruption [11]. Therefore, genes paralogous to *PKP2* were queried from Ensembl 86.

The set of genes that meet all five criteria was named the *PKP2-related genes*. Analogous to the Fam1 and Fam2 gene sets, *PKP2-*related genes were restricted to those expressed in the heart.

The intersection of Fam1 and Fam2 genes with the *PKP2-*related genes was prioritized using the Dintor MetaRanker tool [36], by equally weighting gene expression, number of variants, variant class (consequence class “high” was rated higher than class “moderate”), minimum ExAC allele frequency of the gene’s variant(s), minimum PROVEAN score of the gene’s variant(s), binary prediction (neutral or deleterious) of this PROVEAN score, and presence in a linkage region (see Fig. 2). The LR.PFS3 model [24] was not used in the ranking since it is not defined for indels.

## Results

### Whole exome sequencing

For each of the eight samples relevant to this study (Fam1.I.2, Fam1.II.2, Fam1.III.1, Fam1.III.2, Fam1.III.3, Fam2.I.2, Fam2.II.1, and Fam2.II.2), an average of 54.3 ± 15.3 million reads was generated. The mean base quality was well above 30Q at all read positions, yet a drop in quality could be observed in the last 20 bases of each read. Nearly all reads (99.9%) could be successfully mapped to the human reference genome GRCh37, resulting in a mean coverage of 24.4 ± 3.9X at a mean mapping quality of 45.2 ± 0.8Q. On average, 85.1 ± 3.8% of the exonic target region was covered with at least 10X.

### Identification of candidate genes

The filtering strategy described in the Methods Section and visualized in Fig. 2 resulted in 85 variants in 74 distinct genes in Fam1 and 242 variants in 212 distinct genes in Fam2. The gene sets for both families obtained by this filtering strategy are available as Additional file 1: Table S1. No CNVs in either family met the selection criteria. Since the number of genes per family was too large to analyze in detail, we decided to restrict our analysis to known ACM related genes and then to *PKP2-*related genes.

**Fig. 3:**
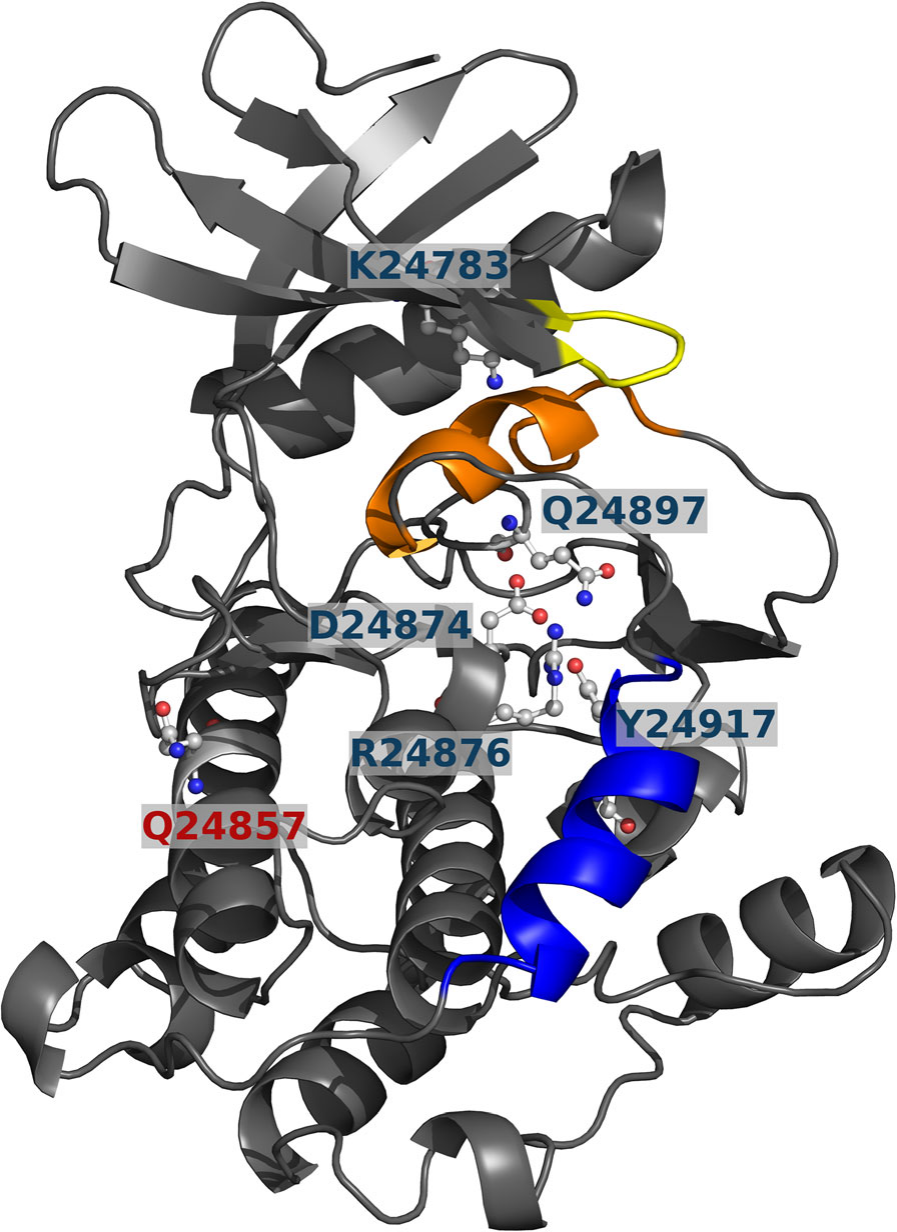
Structure of the titin kinase domain (PDB 1tki chain A). Functional residues are represented as ball and stick, D24874 is the catalytic aspartate. The ATP binding site includes residue K24783 as well as the nearby yellow loop. The calcium/calmodulin binding helix is colored blue, the helix in orange blocks the ATP binding site in this autoinhibited conformation. Residue Q24857 is solvent exposed on the side opposite to the functional residues

From the 15 ACM-related genes, none was present in the gene set of Fam1, while *TTN* was in the gene set of Fam2 (Table 2). Specifically, the affected male patient Fam2.II.1 and his healthy father Fam2.I.1 harbored three distinct heterozygous missense variants in *TTN*. The two rare variants ENSP00000434586.1:p.Gln24857His (isoform N2B) and ENSP00000434586.1:p.Arg23483His (isoform N2B) were predicted deleterious by PROVEAN and LR.PFS3. Both variants were confirmed by Sanger sequencing (see Additional file 1). The third *TTN* variant ENSP00000434586.1:p.Ile3716Val (isoform N2B) is common, not conserved, and was predicted neutral by PROVEAN and LR.PFS3. *TTN* encodes for titin, the largest human protein, which has over 300 highly repetitive independently folding domains, including 152 immunoglobulin like, 132 fibronectine 3 (Fn3), 19 Kelch, 14 tetratricopeptide repeat, and 15 solenoid domains [37]. The variant Arg23483His is located in the 125th of the 132 Fn3 domains (PF00041), while Gln24857His is located inside titin’s only serine kinase domain (PF00069), a structurally conserved protein domain that plays an important role in the regulation of cell proliferation, apoptosis and cell differentiation. The location of this variant and other functional residues in the protein structure of the kinase domain is visualized in Fig. 3. The mutated residue Gln24857His is located opposite of the active site and results in a charge and polarity change. The variant was further predicted to destabilize the protein structure by MAESTROweb (ΔΔG = 1.433, confidence = 0.8). In addition, the wild type residue glutamine was highly conserved in a multiple sequence alignment of homologous sequences from 63 species computed by ConSurf.

**Table 2:** Fam1 and Fam2 genes and corresponding variants that overlap with the ACM genes or the *PKP2-*related genes

Family	Gene set^a^	Gene rank^b^	Gene name	HGVSp	Variant consequence	PROVEAN prediction^c^	Population frequency^d^	Genotype^e^	Comment
Fam2	ACM	–	TTN	ENSP00000434586.1:p.Gln24857His	missense	deleterious	rare	het in Fam2.II.1 and Fam2.I.1	Mutations in TTN can cause ACM [8].
Fam2	ACM	–	TTN	ENSP00000434586.1:p.Arg23483His	missense	deleterious	rare	het in Fam2.II.1 and Fam2.I.1	Mutations in TTN can cause ACM [8].
Fam2	ACM	–	TTN	ENSP00000434586.1:p.Ile3716Val	missense	neutral	common	het in Fam2.II.1 and Fam2.I.1	Mutations in TTN can cause ACM [8].
Fam1	PKP2 (GO: neg. Reg. cell prolif.)	1	DAG1	ENSP00000312435.2:p.Leu86Phe	missense	neutral	rare	het in Fam1.III.2, Fam1.III.3, Fam1.II.1	β-dystroglycan binds to Hippo pathway effector Yap to inhibit cardiomyocyte proliferation in mice [42].
Fam1	PKP2 (GO: heart developm.)	2	TCF25	ENSP00000263347.7:p.Ser390Phe	missense	neutral	rare	het in Fam1.III.2, Fam1.III.3, Fam1.II.1	Negatively regulates SRF, whose increased expression causes cardiomyopathy in mice [43].
Fam1	PKP2 (GO: neg. Reg. cell prolif.)	3	DAB2IP	ENSP00000259371.2:p.Asp10Gly	missense	neutral	rare	het in Fam1.III.2, Fam1.III.3, Fam1.II.1	One variant in DAB2IP has been associated with coronary heart disease [44].
Fam1	PKP2 (GO: neg. Reg. cell prolif.)	4	CTBP2	ENSP00000357816.5:p.Gly70Arg	missense	neutral	common	het in Fam1.III.2, Fam1.III.3, Fam1.II.1	Ctbp2-null mice have defective heart morphogenesis. CTBP2 may be a regulator of Wnt-mediated gene expression [45].
Fam2	PKP2 (GO: neg. Reg. cell prolif.)	1	IRF1	ENSP00000384406.1:p.Asn259Ser	missense	deleterious	rare	het in Fam2.II.1 and Fam2.I.1	IRF1 is associated with cancer and a negative regulator of coronary artery smooth muscle cells [41] (OMIM *147575).
Fam2	PKP2 (GO: reg. of bicell. Tight. junction assembly)	2	MYO1C	ENSP00000412197.2:p.Gln766Lys	missense	neutral	rare	het in Fam2.II.1 and Fam2.I.1	OMIM *606538
Fam2	PKP2 (GO: cardiac muscle cell action pot.)	3	DMD	ENSP00000367948.2:p.Arg2151Trp	missense	neutral	common	hemi in Fam2.II.1, Fam2.I.1; het in Fam2.I.2	Recessive mutations in DMD can cause muscle dystrophy (OMIM *300377).
Fam2	PKP2 (GO: heart development)	4	MKKS	ENSP00000382008.2:p.Ile339Val	missense	neutral	rare	het in Fam2.I.2 and Fam2.II.2	Recessive mutations in MKKS can cause Bardet-Biedl syndrome (OMIM *604896).
Fam2	PKP2 (GO: neg. Reg. cell prolif.)	5	NOTCH2	ENSP00000256646.2:p.Asp1327Gly	missense	neutral	common	het in Fam2.I.1 and Fam2.II.1	This variant has been reported causal for Congenital heart disease as compound heterozygote with L2408H, which is absent in Fam2 [51].
Fam2	PKP2 (GO: heart development)	6	PKD1	ENSP00000456672.1:p.Arg198Trp	missense	neutral	rare	het in Fam2.II.1 and Fam2.I.1	Dominant mutations have been associated with polycystic kidney disease (OMIM *601313).
Fam2	PKP2 (PPI / GO: pos. Reg. sodium ion)	7	SCN5A	ENSP00000398962.2:p.His558Arg	missense	neutral	common	het in Fam2.I.1, Fam2.I.2, Fam2.II.2, Fam1.II.3	This variant has been reported causal for isolated conduction disease as compound heterozygote with T215I, which is absent in Fam2 [52].
Fam2	PKP2 (GO: neg. Reg. cell prolif.)	8	MYOCD	ENSP00000341835.4:p.Gln304del	inframe deletion	neutral	common	het in Fam2.II.1 and Fam2.I.1	Cardiac muscle-specific transcriptional coactivator of serum response factor. Mutations have been associated with hypertrophic cardiomypathy (OMIM *606127).
Fam2	PKP2 (PPI)	9	DSC1	ENSP00000257198.5:p.Cys848Phe	missense	neutral	common	het in Fam2.II.1 and Fam2.I.1	Desmosomal protein desmocolin 1 (*OMIM 125643).
Fam2	PKP2 (PPI)	10	DROSHA	ENSP00000339845.3:p.Ser321Leu	missense	neutral	common	hom in Fam2.I.2, Fam2.II.2; het in all others except Fam1.II.2	Ribonuclease III. Mutations have been associated with cancer (OMIM *608828).

^a^ ACM: known ACM genes; PKP2: PKP2 related genes

^b^ *PKP2*-related genes are listed according to their rank from top to bottom

^c^deleterious: PROVEAN score < −2.5; neutral: PROVEAN score > = 2.5

^d^common: ExAC AF > = 0.01; rare: ExAC AF < 0.01 or NA; ExAC: Exome Aggregation Consortium

^e^het: heterozygous; hom: homozygous; hemi: hemizygous. If an individual is not listed, his/her genotype is homozygous reference

In the next analysis step, we identified 311 genes related to *PKP2* following the criteria defined in the Methods section: (i) Five genes form a complex with *PKP2*, (ii) 33 genes interact with *PKP2*, (iii) four genes are transcription factors of *PKP2*, (iv) 275 genes are involved in one of 12 biological processes together with *PKP2*, (v) and three genes are paralogs of *PKP2*. A list of these *PKP2-*related genes is available as Additional file 1: Table S2. Four Fam1 genes and ten Fam2 genes were in the set of *PKP2-*related genes and were prioritized based on their expression in the heart, the number and type of variants, their allele frequencies and deleteriousness prediction. These *PKP2*-related genes and variants of both families are summarized in Table 2. The full table with additional, detailed annotations is available as Additional file 1: Table S3.

Of the four *PKP2-*related genes *DAG1*, *DAB2IP*, and *CTBP2* in Fam1 are associated with *PKP2* through the GO BP process “negative regulation of cell migration”, while *TCF25* is associated with PKP2 through the GO BP process “heart development”. The variants in all four genes are predicted neutral by PROVEAN, and all but the one in *CTBP2* are rare. The highest ranking *PKP2-*related gene in Fam1 is *DAG1*, which encodes for dystroglycan, a central component of the dystrophin–glycoprotein complex (DGC). Dystroglycan is post-translationally cleaved into α- and β-dystroglycan subunits [38]. α-dystroglycan is an extracellular protein involved in the interactions between DGC and extracellular matrix components, while β-dystroglycan contains a single transmembrane domain and a C-terminal cytoplasmic tail. The *DAG1* candidate substitution ENSP00000312435.2:p.Leu86Phe is located in the α-dystroglycan N-terminal region, where the leucine side chain is solvent exposed on the side of a Ig-like domain [39]. The position is in close proximity to Thr63, identified as a O-glycosylation [40] (see Additional file 1: Fig. S1). The variant was predicted to stabilize protein structure by MAESTROweb (ΔΔG = −0.231, confidence = 0.9). For the other three candidate genes, no protein structure was available in PDB, so predictions with MAESTROweb could not be computed.

The highest ranking *PKP2-*related gene in Fam2 is the interferon regulatory factor 1 (*IRF1*), which harbors a rare, deleterious-predicted missense variant in the affected male patient Fam2.II.1 and his healthy father Fam2.I.1. *IRF1* inhibits cell growth in coronary artery smooth muscle cells [41]. Repression of *IRF1* has lead to a higher susceptibility to the formation of neointima (scar tissue) following vessel injury in mice [41].

## Discussion

In this study we investigated the genetic cause of ACM in two families using whole exome sequencing. Since all affected and some unaffected individuals were known to harbor *PKP2* variants, we investigated whether a second gene was involved in a digenic inheritance pattern, with the second gene either causing ACM in the affected individuals together with *PKP2*, or compensating the effect of the *PKP2* variants in the carriers. We identified 74 and 212 genes in families 1 and 2, respectively, which carried variants consistent with the mode of digenic inheritance. To obtain results that can be easily interpreted, we restricted our analysis to genes either associated with ACM or related to *PKP2.* In family 1 we identified four genes that are annotated with the same biological process as *PKP2*. In family 2 we identified the ACM associated gene *TTN* and ten genes related to *PKP2* through a shared biological process or protein interactions (see Fig. 2).

Of the four *PKP2-*related Fam1 genes, the genes homologous to *DAG1*, *TCF25*, and *CTBP2* have been linked to cardiomyocyte proliferation or heart development in mice and, in case of *TCF25*, also in human. *DAG1* and *TCF25* negatively regulate heart development, while a knock out of *CTBP2* leads to a lethal malformation of the heart in mice. A variant in *DAB2IP* has been associated with coronary heart disease in two studies, indicating that this gene might also play a crucial rule for the normal functioning of the heart. It has been reported that β-dystroglycan, a protein product of *DAG1*, directly binds to the Hippo pathway effector Yap to inhibit cardiomyocyte proliferation in mice [42]. In particular, the Hippo pathway and DGC cooperatively regulate tissue growth in mouse hearts after injury. Yap and the Hippo pathway have been directly implicated in ACM pathogenesis [30]. *TCF25* (previously named *NULP1*) was suggested as a transcription factor that negatively regulates the serum response factor (SRF). SRF controls muscle differentiation and cellular growth and regulates cardiac genes. SRF over-expression has been shown to cause cardiomyopathy and cardiac hypertrophy in mice. Therefore, *TCF25* may function as a transcriptional repressor of SRF in human heart development [43]. *DAB2IP* acts as a tumor suppressor gene, and is inactivated by methylation in prostate and breast cancers. A genome-wide association study found the rs7025486 variant in *DAB2IP* associated with coronary heart disease, which was replicated in a second study [44]. *CTBP2* encodes two proteins, a transcriptional repressor and a major component of synaptic ribbons. Silencing the homologous *Ctbp2* gene in mice causes defects in heart morphogenesis and results in early embryonic lethality [45]. *Ctbp2*-null mice show similar axial truncation phenotypes as mice with mutations in some Wnt target genes, suggesting that CTBP2 may be a regulator of Wnt-mediated gene expression [45]. Indeed, CtBP2 acts as corepressor of C/EBPα, an early regulator of adipogenesis, and target of the Wnt signaling pathway [46]. Furthermore, *Sox6* has been found to bind *Ctbp2* to repress the fibroblast growth factor 3 [47] and *Sox6* to regulate the cardiac myocyte development in mice [48]. Although none of the variants in these four genes are predicted to be deleterious and the variant in *DAG1* is even predicted to stabilize protein structure, they could nevertheless affect protein stability, flexibility, and interaction with the other binding partners. However, in the present study we could not find any indication that these genes may act together with *PKP2* to cause the ACM phenotype in the affected individuals of Fam1.

Since *TTN* is a known ACM associated gene, it is a likely candidate in the second family. *TTN* encodes for titin, the largest human protein with isoforms ranging from about 27.000 to 36.000 amino acids. Titin is functionally linked to the desmosome (and thereby to *PKP2*), since titin filaments are a key component of sarcomeres and connect to the transitional junction at the intercalated disk [8]. In a cohort of 38 ACM families, Taylor et al. identified novel *TTN* mutations in 18% of the families [8]. In addition to ACM, *TTN* has been associated with dilated, hypertrophic, and restrictive cardiomyopathy [49]; its association with hypertropic cardiomyopathy, however, is still under debate [50]. The affected patient Fam2.II.1 and his father both harbor two rare heterozygous missense variants that are predicted deleterious. The Gln24857His variant is located in titin’s only kinase domain at a conserved position and is predicted to destabilize protein structure, while Arg23483His is located in one of the 132 Fn3 domains. Therefore, the Gln24857His variant is more likely to impair titin function than the Arg23483His variant, even though disease causal variants in repetitive titin domains have been reported [8]. Studies with transfected cell lines have shown that heterozgyous mutations, in contrast to homozygous mutations, still allow for functional sarcomeres but may alter the organizational characteristics and impair the normal cardiac function [49]. These findings agree well with the hypothesis that either one or both of these variants alter the structure of titin and only lead to ACM in combination with the *PKP2* mutation.

In addition to the genes described here in more detail, there are other promising candidates for the second causal gene in Fam2 (see Table 2). For example, of the *PKP2-*related genes, *NOTCH2* and *SCN5A* harbor one of two compound heterozygous variants that have been reported to cause congenital heart disease and isolated conduction disease, respectively [51, 52]; *DMD* harbors a neutral hemizygous variant in the affected Fam2 individual, a gene where recessive variants can cause muscle dystrophy; *DAG1* and *MKKS* are associated with recessive diseases, yet the variants in the affected individuals are heterozygous; *IRF1* is associated with non-cardiac diseases; *DSC1*, a desmosomal gene not associated with heart disease, harbors a common missense variant that is predicted neutral. Since all of these genes are interesting candidates for follow up studies, it would be interesting to test whether the same or other rare variants in our candidate genes can be identified in a large cohort ACM patients, both in patients carrying desmosomal mutations or other ACM related mutations as well as in genetically unsolved cases.

Digenic inheritance has previously been reported as a disease-causal mechanism for ACM, however, these studies have focused on desmosomal genes. As a result, there are no reports of digenic inheritance in ACM with *PKP2* and a non-desmosomal gene such as *TTN*.

We are aware of limitations in our study. Both families have relatively few members, which resulted in a large set of variants and genes as possible candidates for digenic inheritance. Only 85% of the exome was covered with at least 10X. Since we required a minimum coverage of 10X to accept a variant call, 15% of the exome could not be investigated. However, coverage at the ACM genes was well above average, so it is unlikely that variants were missed in these genes. Due to the large number of candidate genes in each family, we restricted our analysis to ACM or *PKP2-*related genes, potentially removing causative digenic genes with unknown associations. Even though the *TTN* variants in Fam2 were confirmed by Sanger sequencing, no functional validation of the variant effects was performed. Therefore, it still needs to be shown if *TTN* or any of the candidate genes in Fam1 truly cause ACM together with *PKP2* in the respective family. A functional validation could be performed based on induced pluripotent stem cell (iPSC) models from the ACM-affected individuals, where the *PKP2* variant or the *TTN*/Fam1 variants are reversed [53]. In the iPSC derived cardiomyocytes the effect of the genetic variants could be investigated by comparing fat accumulation and cell electrophysiology to the double mutant cells. Other cell models that could be employed for validation are progenitor cells (they differentiate easily in vitro), non-contractile cardiac mesenchymal stromal cells (ideal for studying lipid metabolism), or primary or immortalized cardiomyocytes (enable investigation of gap-junctions and ion-channels) [54]. Yet, even if successful, such experiments would demonstrate the mode of effect in the respective family, while general conclusions about the role of *TTN*/Fam1 genes in ACM could not necessarily be drawn from them. To evaluate the roles of these genes in ACM more generally, other ACM patients carrying desmosomal variants could be checked for rare variants in the respective genes. Unfortunately, we currently do not have additional ACM patients for testing and the NCBI Sequence Read Archive does not contain public whole exome or whole genome sequence data of ACM patients. The search for genes with a digenic effect is a considerable challenge since variants in both relevant genes do not necessarily have a pathogenic effect when occurring individually [11]. The functional or structural change caused by the variant in either protein may be subtle, and may for example lead to a change at a protein binding affinity or a change in gene expression. Therefore, standard criteria usually applied to evaluate the likelihood of variant pathogenicity like rarity and computational predictions might not be well suited. Consequently, we did not exclude variants based on these criteria, yet in the absence of functional validation and more appropriate models, we prioritized and discussed our results according to these methods. We would like to recall that we did not distinguish between variants that were present in the affected and not in the carriers and variants present in the carriers but not in the affected, since we were interested in genes whose function might differ between affected individuals and carriers due to the variants. However, we point out that of the 17 variants listed in Table 2, only three (Ile339Val in *MKKS*, His558Arg in *SCN5A*, and Ser321Leu in *DROSHA*) are present in the carriers and not in the affected individuals, suggesting that our strategy of prioritizing based on rarity and predicted pathogenicity is appropriate. Finally, we acknowledge the possibility that more than two genes could be involved in the pathogenesis (oligogenic inheritance) or, contrarily, that environmental factors could influence the penetrance of the *PKP2* variants without other genetic variants having an effect on the development of ACM. However, in our study, the main non-genetic disease modulators (age, sex, and physical exercise) are not sufficient to explain the different phenotypic expression in affected individuals and carriers in the two analyzed families (see Table 1). In Fam1, both the carrier Fam1.III.1 and the affected Fam1.III.2 are male and are close in age, and carrier Fam1.III.1 and the affected Fam1.III.3 are both physically active. In Fam2, both the affected Fam2.II.1 and the carrier Fam2.II.2 are male, physically active, and relatively close in age.

## Conclusions

In the present work we have provided further indication that a single (desmosomal) mutation might not be sufficient to cause ACM, by showing the co-segregation of other variants with *PKP2* and the phenotype in two families.

## Additional file

Additional file 1: Includes Supplementary Methods; Tables S1, S2, and S3; Fig. S1. (DOCX 2344 kb)

## Abbreviations

ACM: Arrhythmogenic cardiomyopathy
ARVC: arrhythmogenic right ventricular cardiomyopathy
BP: biological process
CNV: copy number variation
Fam1: family 1
Fam2: family 2
Fn3: fibronectine 3
GO: Gene Ontology
iPSC: induced pluripotent stem cell
SNP: single nucleotide polymorphism

## References

[1] Ohno S (2016). The genetic background of arrhythmogenic right ventricular cardiomyopathy. J Arrhythmia.

[2] Garcia-Gras E, Lombardi R, Giocondo MJ, Willerson JT, Schneider MD, Khoury DS (2006). Suppression of canonical Wnt/β-catenin signaling by nuclear plakoglobin recapitulates phenotype of arrhythmogenic right ventricular cardiomyopathy. J Clin Invest.

[3] Chen SN, Gurha P, Lombardi R, Ruggiero A, Willerson JT, Marian AJ (2014). The hippo pathway is activated and is a causal mechanism for adipogenesis in arrhythmogenic cardiomyopathy. Circ Res.

[4] Ortiz-Genga MF, Cuenca S, Dal Ferro M, Zorio E, Salgado-Aranda R, Climent V, Padrón-Barthe L (2016). Truncating FLNC mutations are associated with high-risk dilated and Arrhythmogenic cardiomyopathies. J Am Coll Cardiol.

[5] Mayosi BM, Fish M, Shaboodien G, Mastantuono E, Kraus S, Wieland T, et al. Identification of cadherin 2 (CDH2) mutations in Arrhythmogenic right ventricular cardiomyopathy. Circ Cardiovasc Genet. 2017;10(2) 10.1161/CIRCGENETICS.116.001605 28280076

[6] Sen-Chowdhry S, Syrris P, Pantazis A, Quarta G, McKenna WJ, Chambers JC (2010). Mutational heterogeneity, modifier genes, and environmental influences contribute to phenotypic diversity of arrhythmogenic cardiomyopathy. Circ Cardiovasc Genet.

[7] Severini GM, Krajinovic M, Pinamonti B, Sinagra G, Fioretti P, Brunazzi MC, Falaschi A, Camerini F, Giacca M, Mestroni LA (1996). New locus for Arrhythmogenic right ventricular dysplasia on the long arm of chromosome 14. Genomics.

[8] Taylor M, Graw S, Sinagra G, Barnes C, Slavov D, Brun F (2011). Genetic variation in titin in arrhythmogenic right ventricular cardiomyopathy-overlap syndromes. Circulation.

[9] Li D, Ahmad F, Gardner MJ, Weilbaecher D, Hill R, Karibe A (2000). The locus of a novel gene responsible for Arrhythmogenic right-ventricular dysplasia characterized by early onset and high penetrance maps to chromosome 10p12-p14. Am J Hum Genet.

[10] Andreasen C, Nielsen JB, Refsgaard L, Holst AG, Christensen AH, Andreasen L (2013). New population-based exome data are questioning the pathogenicity of previously cardiomyopathy-associated genetic variants. Eur J Hum Genet.

[11] Cooper DN, Krawczak M, Polychronakos C, Tyler-Smith C, Kehrer-Sawatzki H (2013). Where genotype is not predictive of phenotype: towards an understanding of the molecular basis of reduced penetrance in human inherited disease. Hum Genet.

[12] Xu T, Yang Z, Vatta M, Rampazzo A, Beffagna G, Pillichou K (2010). Compound and Digenic heterozygosity contributes to Arrhythmogenic right ventricular cardiomyopathy. J Am Coll Cardiol.

[13] Rasmussen TB, Palmfeldt J, Nissen PH, Magnoni R, Dalager S, Jensen UB (2013). Mutated Desmoglein-2 proteins are incorporated into desmosomes and exhibit dominant-negative effects in Arrhythmogenic right ventricular cardiomyopathy. Hum Mutat.

[14] Nakajima T, Kaneko Y, Irie T, Takahashi R, Kato T, Iijima T, Iso T, Kurabayashi M (2012). Compound and digenic heterozygosity in desmosome genes as a cause of arrhythmogenic right ventricular cardiomyopathy in Japanese patients. Circ J.

[15] Génin E, Feingold J, Clerget-Darpoux F (2008). Identifying modifier genes of monogenic disease: strategies and difficulties. Hum Genet.

[16] Meyer S, van der Meer P, van Tintelen JP, van den Berg MP (2014). Sex differences in cardiomyopathies. Eur J Heart Fail.

[17] James CA, Bhonsale A, Tichnell C, Murray B, Russell SD, Tandri H (2013). Exercise increases age-related penetrance and arrhythmic risk in arrhythmogenic right ventricular dysplasia/cardiomyopathy-associated desmosomal mutation carriers. J Am Coll Cardiol.

[18] Marcus FI, McKenna WJ, Sherrill D, Basso C, Bauce B, Bluemke DA (2010). Diagnosis of arrhythmogenic right ventricular cardiomyopathy/dysplasia. Eur Heart J.

[19] Li H, Durbin R (2009). Fast and accurate short read alignment with burrows-wheeler transform. Bioinformatics.

[20] McKenna A, Hanna M, Banks E, Sivachenko A, Cibulskis K, Kernytsky A (2010). The genome analysis toolkit: a MapReduce framework for analyzing next-generation DNA sequencing data. Genome Res.

[21] Kersey PJ, Allen JE, Armean I, Boddu S, Bolt BJ, Carvalho-Silva D (2016). Ensembl genomes 2016: more genomes, more complexity. Nucleic Acids Res.

[22] Lek M, Karczewski KJ, Samocha KE, Banks E, Fennell T, AH O, et al. Analysis of protein-coding genetic variation in 60,706 humans. Nature. 2016:285–91. 10.1038/nature19057 PMC5018207 27535533

[23] Choi Y, Sims GE, Murphy S, Miller JR, Chan AP (2012). Predicting the functional effect of amino acid substitutions and Indels. PLoS One.

[24] König E, Rainer J, Domingues FS (2016). Computational assessment of feature combinations for pathogenic variant prediction. Mol Genet Genomic Med.

[25] Fromer M, Purcell SM (2014). Using XHMM software to detect copy number variation in whole-exome sequencing data. Curr Protoc Hum Genet.

[26] Laimer J, Hofer H, Fritz M, Wegenkittl S, Lackner P (2015). MAESTRO--multi agent stability prediction upon point mutations. BMC Bioinformatics.

[27] Laimer J, Hiebl-Flach J, Lengauer D, Lackner P (2016). MAESTROweb: a web server for structure-based protein stability prediction. Bioinformatics.

[28] Glaser F, Pupko T, Paz I, Bell RE, Bechor-Shental D, Martz E (2003). ConSurf: identification of functional regions in proteins by surface-mapping of phylogenetic information. Bioinformatics.

[29] Uhlén M, Fagerberg L, Hallström BM, Lindskog C, Oksvold P, Mardinoglu A (2015). Tissue-based map of the human proteome. Science.

[30] Rampazzo A, Calore M, Van Hengel J, Van Roy F (2014). Intercalated discs and arrhythmogenic cardiomyopathy. Circ Cardiovasc Genet.

[31] Huttlin EL, Ting L, Bruckner RJ, Gebreab F, Gygi MP, Szpyt J (2015). The BioPlex network: a systematic exploration of the human interactome. Cell.

[32] Szklarczyk D, Franceschini A, Wyder S, Forslund K, Heller D, Huerta-Cepas J (2014). STRING v10: protein--protein interaction networks, integrated over the tree of life. Nucleic Acids Res.

[33] Calderone A, Castagnoli L, Cesareni G (2013). Mentha: a resource for browsing integrated protein-interaction networks. Nat Meth.

[34] Lesurf R, Cotto KC, Wang G, Griffith M, Kasaian K, Jones SJM (2016). The open regulatory annotation consortium. ORegAnno 3.0: a community-driven resource for curated regulatory annotation. Nucleic Acids Res.

[35] Gene Ontology Consortium (2015). Gene ontology consortium: going forward. Nucleic Acids Res.

[36] Weichenberger CX, Blankenburg H, Palermo A, D’Elia Y, König E, Bernstein E (2015). Dintor: functional annotation of genomic and proteomic data. BMC Genomics.

[37] Chauveau C, Rowell J, Ferreiro AA (2014). Rising titan: TTN review and mutation update. Hum Mutat.

[38] Barresi R (2006). Dystroglycan: from biosynthesis to pathogenesis of human disease. J Cell Sci.

[39] Covaceuszach S, Bozzi M, Bigotti MG, Sciandra F, Konarev PV, Brancaccio A (2017). Structural flexibility of human α-dystroglycan. FEBS Open Bio.

[40] Halim A, Rüetschi U, Larson G, Nilsson J (2013). LC-MS/MS characterization of O-glycosylation sites and glycan structures of human cerebrospinal fluid glycoproteins. J Proteome Res.

[41] Wessely R, Hengst L, Jaschke B, Wegener F, Richter T, Lupetti R (2003). A central role of interferon regulatory factor-1 for the limitation of neointimal hyperplasia. Hum Mol Genet.

[42] Morikawa Y, Heallen T, Leach J, Xiao Y, Martin JF (2017). Dystrophin–glycoprotein complex sequesters yap to inhibit cardiomyocyte proliferation. Nature.

[43] Cai Z, Wang Y, Yu W, Xiao J, Li Y, Liu L (2006). Hnulp1, a basic helix-loop-helix protein with a novel transcriptional repressive domain, inhibits transcriptional activity of serum response factor. Biochem Biophys Res Commun.

[44] Harrison SC, Cooper JA, Li K, Talmud PJ, Sofat R, Stephens JW (2012). Association of a sequence variant in DAB2IP with coronary heart disease. Eur Heart J.

[45] Chinnadurai G (2003). CtBP family proteins: more than transcriptional corepressors. BioEssays.

[46] Vernochet C, Peres SB, Davis KE, McDonald ME, Qiang L, Wang H (2009). C/EBP and the corepressors CtBP1 and CtBP2 regulate repression of select visceral white adipose genes during induction of the Brown phenotype in white adipocytes by peroxisome proliferator-activated receptor agonists. Mol Cell Biol.

[47] Murakami A, Ishida S, Thurlow J, Revest JM, Dickson C (2001). SOX6 binds CtBP2 to repress transcription from the Fgf-3 promoter. Nucleic Acids Res.

[48] Cohen-Barak O, Yi Z, Hagiwara N, Monzen K, Komuro I, Brilliant MH (2003). Sox6 regulation of cardiac myocyte development. Nucleic Acids Res.

[49] Neiva-Sousa M, Almeida-Coelho J, Falcão-Pires I, Leite-Moreira AF (2015). Titin mutations: the fall of goliath. Heart Fail Rev.

[50] Herman DS, Lam L, Taylor MRG, Wang L, Teekakirikul P, Christodoulou D (2012). Truncations of titin causing dilated cardiomyopathy. N Engl J Med.

[51] Priest JR, Osoegawa K, Mohammed N, Nanda V, Kundu R, Schultz K (2016). De novo and rare variants at multiple loci support the Oligogenic origins of atrioventricular septal heart defects. PLoS Genet.

[52] Viswanathan PC, Benson DW, Balser JRA (2003). Common SCN5A polymorphism modulates the biophysical effects of an SCN5A mutation. J Clin Invest.

[53] Hockemeyer D, Jaenisch R (2016). Induced pluripotent stem cells meet genome editing. Cell Stem Cell.

[54] Sommariva E, Stadiotti I, Perrucci GL, Tondo C, Pompilio G (2017). Cell models of arrhythmogenic cardiomyopathy: advances and opportunities. Dis Model Mech.

