# Supplementary material for: Exploring digenic inheritance in arrhythmogenic cardiomyopathy
Source: BMC Med Genet. 2017 Dec 8;18:145. doi: 10.1186/s12881-017-0503-7 (PMC5723071; doi:10.1186/s12881-017-0503-7)
Supplement: Additional file 1: — Includes Supplementary Methods; Tables S1, S2, and S3; Fig. S1. (DOCX 2344 kb) [file 12881_2017_503_MOESM1_ESM.docx]

**Supplementary Methods**

**Subjects**

Family 1 (Fam1) consists of eight individuals in three generations of which two are affected by ACM (Fam1.III.2 and Fam1.III.3). In 2012 the department of Cardiac, Thoracic and Vascular Sciences at Padua University in Padua, Italy has performed denaturing high pressure liquid chromatography or direct Sanger sequencing on the exons and the intronic flanking regions of the genes *DSG2* (NM_001943), *PKP2* (NM_004572), *DSP* (NM_004415), *DSC2* (NM_024422), and *JUP* (NM_002230) in all Fam1 individuals except Fam1.I.2. Exons showing a non-reference pattern were again Sanger sequenced. In *PKP2*, the heterozygous one base-pair deletion *NM_004572.3(PKP2)*:c.2013delC, *NP_004563.2(PKP2)*:p.Lys672ArgfsTer12, which results in a premature stop codon after a frameshift mutation, was identified in the four individuals Fam1.II.2, Fam1.III.1, Fam1.III.2, and Fam1.III.3. The variation was confirmed by next-generation sequencing and by Sanger sequencing (see paragraph *Sanger validation* below) in the four individuals and in Fam1.I.2, for which no previous analysis was performed (see Figure 1).

Family 2 (Fam2) consists of two parents and their two sons of which the son Fam2.II.1 is affected by ACM. In 2013, the medical laboratory of the Niguarda hospital in Milan, Italy performed Sanger sequencing on the exons and the intronic flanking regions of *PKP2* (NG_009000.1) in the patient Fam2.II.1, where the heterozygous deletion *NG_009000.1(PKP2)*:c.2569_2577+41del*,* was identified. This variation describes a deletion crossing an exon / intron border. In-house Sanger sequencing (see paragraph *Sanger validation* below) confirmed this deletion in the Fam2.II.1, yet it was additionally identified in the patient's mother Fam2.I.2 and brother Fam2.II.2, but not in the father Fam2.I.1 (see Figure 1).

**Data generation**

Samples were quantified with the Nanodrop spectrophotometer (Thermo Fischer Scientific) and diluted to approximately 10 ng/µl. The concentration was measured with Quantifluor dsDNA fluorescent DNA-binding dye (#E2670, Promega) on a Qbit Fluorometer (Invitrogen) and adjusted to 10 ng/µl. 100 ng of each sample was tagmented, amplified, and purified following the Nextera Rapid Capture Exome and Expanded Exome Enrichment kit protocol (#FC-140-1003 , Illumina). Two times eight quantified double-indexed samples were pooled together, hybridized with coding exome oligos, captured, amplified, and purified according to protocol. The library pools were quantified with Quantifluor dsDNA fluorescent DNA-binding dye and library size was evaluated by capillary electrophoresis on an Experion Automated Electrophoresis Station (BioRad). Each of the two pools was sequenced on one lane of a HiSeq 2500 in paired end mode with a read length of two times one hundred base pairs.

**Data processing**

For each sample, raw read quality was controlled with FastQC version 0.11.3. Illumina adapters were trimmed off the reads with SeqPrep (https://github.com/jstjohn/SeqPrep), and the success of the clipping was again controlled with FastQC. Reads were aligned to the human reference genome GRCh37 with BWA version 0.7.12 [1]. Alignment quality was examined with QualiMap version v2.1.2 [2] and custom R scripts. Next, PCR duplicates were marked with the Picard MarkDuplicates program (http://broadinstitute.github.io/picard), followed by indel realignment and base quality score recalibration with GATK 3.4-46 [3], following the GATK best practice recommendations [4,5]. The quality of the final alignment was again controlled with QualiMap. Coverage of the X and Y chromosome was inspected to determine inter gender sample swaps. The verifyBamID program [6] was used to determine possible contamination of samples with foreign DNA. For each per-sample alignment, an intermediate genomic gVCF was computed using the GATK HaplotypeCaller. Joint genotype calling was performed with GATK GenotypeGVCFs on all 12 samples and 56 additional samples unrelated to this study to enhance the power of the variant calling. The raw multi-sample variant call set was recalibrated using the GATK variant quality score recalibration tools, following the best practice recommendations. Quality statistics of the final call set was controlled using the GATK VariantEval tool and custom R scripts. Finally, the multi-sample VCF file was subset to include only the 12 samples from this study. Variants were annotated with genomic information from the Ensembl database, including variant consequences in the transcripts, SIFT [7], PolyPhen-2 [8], and Gerp [9] scores using the Dintor tool-suite version V-2014-08 [10]. Population allele frequencies (AF) were retrieved from the ExAC project [11]. We call a variant *rare* if it has an ExAC AF smaller 0.01, and *conserved* if it has a Gerp score greater 3. PROVEAN scores v1.1 were retrieved to serve as a method for variant deleteriousness prediction, since PROVEAN scores are defined for both SNPs and indels [12]. PROVEAN scores smaller -2.5 are classified as deleterious [12]. However, combining PROVEAN predictions with PON-P2 and GO.BP has been shown to outperform PROVEAN for SNP prediction [13]. Therefore, the logistic regression model trained on these three features as defined in [13] was used to obtain predictions for all SNPs. The model, named LR.PFS3 (for logistic regression on predictive feature set 3), produces a prediction value between 0 and 1 and classifies SNPs with a score greater 0.525 as pathogenic. Genes were further annotated with a binary flag to indicate if they were located in one of the three ACM linkage regions ARVD3:14q12-q24, ARVD4:2q32.1-q32.3, or ARVD6:10p14-p12.

Copy number variations (CNVs) were called with the program XHMM [14], following the authors recommendations and suggested parameters and filtering steps [15]. Finally, CNVs were filtered to those with a quality score (Q_SOME) greater Q60, using the same threshold as the authors [14].

**Details on the family-based gene selection**

Additionally to the individuals of the two families Fam1 and Fam2, an unrelated female ACM patient carrying an exon 4 PKP2 deletion (indiv.A), her healthy sister also carrying the PKP2 deletion (indv.B), and their healthy aunt also carrying the PKP2 deletion (indv.C) were used to select genes harboring variant(s) with a specific genotype in the family members of Fam1 and Fam2. Specifically, the selection of point (ii) of the Methods subsection “Family-based gene selection” was performed as follows.

Selection for Fam1: The variant is present in Fam1.III.2 and Fam1.III.3 (all affected of Fam1) and not present in Fam1.III.1, Fam1.II.2, Fam1.I.2, Fam2.I.2, Fam2.II.2, indv.B, indv.C (all healthy carriers) OR the variant is present in Fam1.III.1, Fam1.II.2, Fam1.I.2 (all healthy carriers of Fam1) and not present in Fam1.III.2, Fam1.III.3, Fam2.II.1, indv.A (all affected carriers).

Selection for Fam2: The variant in present in Fam2.II.1 (all affected of Fam2), but absent in Fam2.I.2, Fam2.II.2, Fam1.III.1, Fam1.II.2, Fam1.I.2, indv.B, indv.C (all healthy carriers) OR the variant is present in Fam2.I.2 and Fam2.II.2 (all healthy carriers of Fam2), but absent in Fam2.II.1, Fam1.III.2, Fam1.III.3, indv.A (all affected carriers).

**Sanger validation**

Variants were validated by Sanger sequencing using a combined set of forward and reverse primers. The main PCR conditions used were: 95°C 5 minutes/ 98°C 20 seconds; °C 20 seconds (melting temperature chance based on the primers) TM; 72°C 30 seconds 25 cycles/72 °C 1 minute 30 seconds ;4°C 10 minutes; 16°C ∞, in 5 $\mu$l reaction. All reactions were performed on an Eppendorf pro S instrument. The amplification product was purified using the QIAquick 96 PCR Purification Kit (28181) and PCR product size was inspected using agarose-gel electrophoresis 1%. For the Fam2 *NG_009000.1(PKP2)*:c.2569_2577+41del mutation, PCR products were run on a 2% agarose gel, the bands were excised, the DNA extracted (Qiaquick gel extraction kit) and the two bands were separately sequenced to clearly establish the genotypes. Both forward and reverse DNA strands were sequenced with the Sanger method, using the conditions 95°C 4 minutes/ 95°C 30 seconds; 60°C 4 minutes 28 cycles; 60°C 7 minutes 4°C 10 minutes; 16°C. The sequencing products were run using the capillary ABI 3730 analyser (Applied Biosystems).


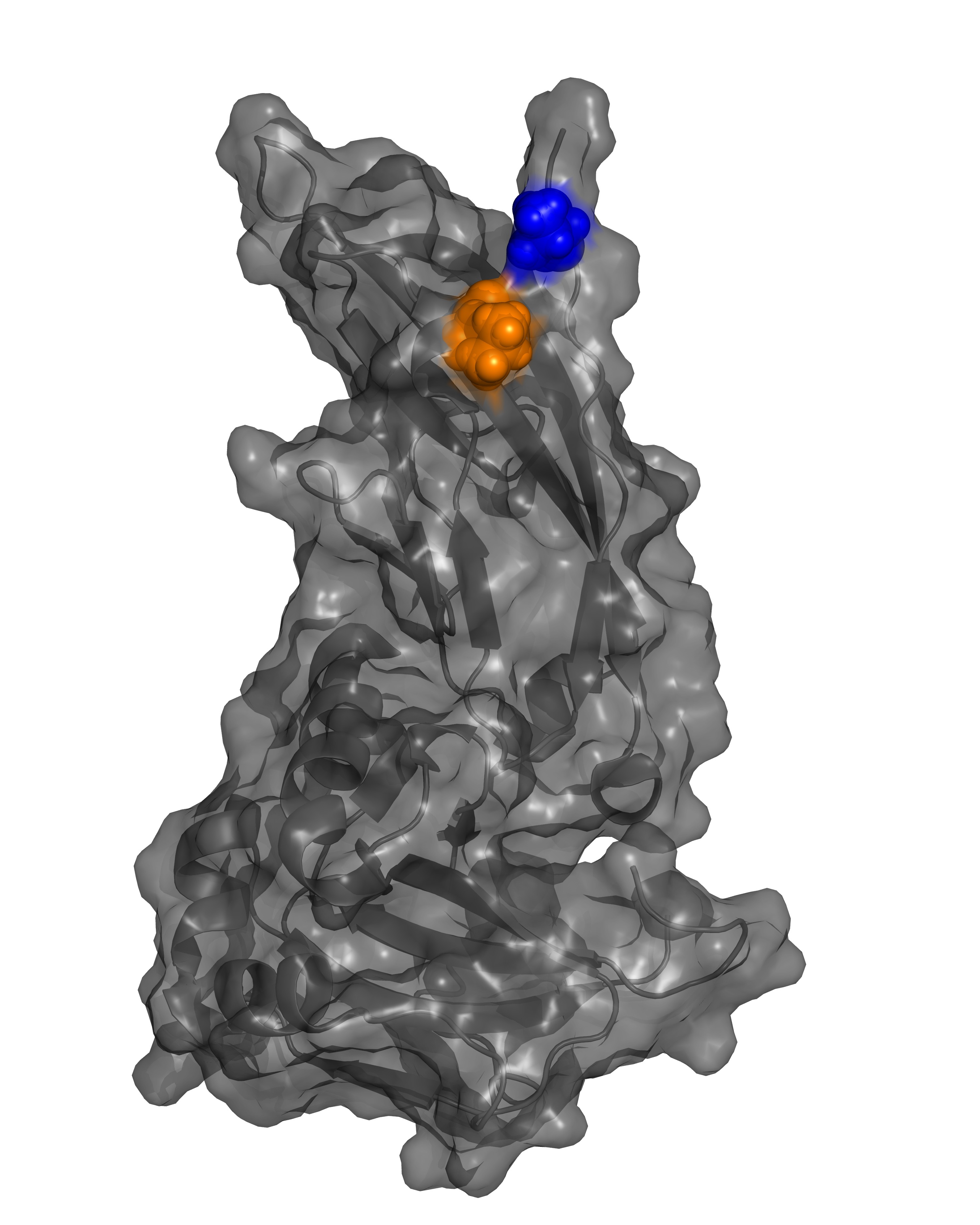
**Supplementary Figure S1.** Cartoon and surface representation of α-dystroglycan N-terminal region. Candidate leucine 86 in orange, O-glycosylation site at threonine 63 in blue. Based on PDB entry 5LLK chain A [24].

**Supplementary Table S1.**

| **Family** | **Ensembl Gene ID** | **HGNC gene name** | **UniProt ID** | **Entrez gene ID** |
| --- | --- | --- | --- | --- |
| Fam1 | ENSG00000114353 | GNAI2 |  | 2771 |
| Fam1 | ENSG00000114098 | ARMC8 | Q8IUR7 | 25852 |
| Fam1 | ENSG00000183853 | KIRREL |  | 101928229 |
| Fam1 | ENSG00000173402 | DAG1 |  | 1605 |
| Fam1 | ENSG00000004534 | RBM6 | P78332 | 10180 |
| Fam1 | ENSG00000144747 | TMF1 | P82094 | 7110 |
| Fam1 | ENSG00000163607 | GTPBP8 | Q8N3Z3 | 29083 |
| Fam1 | ENSG00000182923 | CEP63 | Q96MT8 | 80254 |
| Fam1 | ENSG00000160746 | ANO10 | Q9NW15 | 55129 |
| Fam1 | ENSG00000145860 | RNF145 | Q96MT1 | 153830 |
| Fam1 | ENSG00000136848 | DAB2IP |  | 153090 |
| Fam1 | ENSG00000119392 | GLE1 | Q53GS7 | 2733 |
| Fam1 | ENSG00000167136 | ENDOG | Q14249 | 2021 |
| Fam1 | ENSG00000111799 | COL12A1 |  | 1303 |
| Fam1 | ENSG00000189320 | FAM180A | Q6UWF9 | 389558 |
| Fam1 | ENSG00000136861 | CDK5RAP2 | Q96SN8 | 55755 |
| Fam1 | ENSG00000128567 | PODXL |  | 5420 |
| Fam1 | ENSG00000105983 | LMBR1 | Q8WVP7 | 64327 |
| Fam1 | ENSG00000019144 | PHLDB1 | Q86UU1 | 102466719 |
| Fam1 | ENSG00000059122 | FLYWCH1 | Q4VC44 | 84256 |
| Fam1 | ENSG00000109917 | ZNF259 |  | 8882 |
| Fam1 | ENSG00000175029 | CTBP2 | P56545 | 1488 |
| Fam1 | ENSG00000138162 | TACC2 | O95359 | 10579 |
| Fam1 | ENSG00000152558 | TMEM123 | Q8N131 | 114908 |
| Fam1 | ENSG00000197893 | NRAP | Q86VF7 | 4892 |
| Fam1 | ENSG00000102699 | PARP4 | Q9UKK3 | 143 |
| Fam1 | ENSG00000140545 | MFGE8 |  | 4240 |
| Fam1 | ENSG00000101365 | IDH3B | O43837 | 3420 |
| Fam1 | ENSG00000178127 | NDUFV2 |  | 4729 |
| Fam1 | ENSG00000182871 | COL18A1 | P39060 | 80781 |
| Fam1 | ENSG00000124440 | HIF3A |  | 64344 |
| Fam1 | ENSG00000131149 | GSE1 | Q14687 | 23199 |
| Fam1 | ENSG00000183044 | ABAT | P80404 | 18 |
| Fam1 | ENSG00000158545 | ZC3H18 | Q86VM9 | 124245 |
| Fam1 | ENSG00000105223 | PLD3 |  | 23646 |
| Fam1 | ENSG00000141002 | TCF25 |  | 22980 |
| Fam1 | ENSG00000130158 | DOCK6 | Q96HP0 | 57572 |
| Fam1 | ENSG00000132386 | SERPINF1 | P36955 | 5176 |
| Fam1 | ENSG00000198848 | CES1 |  | 100653057 |
| Fam1 | ENSG00000129226 | CD68 | P34810 | 968 |
| Fam1 | ENSG00000105397 | TYK2 |  | 7297 |
| Fam1 | ENSG00000101745 | ANKRD12 |  | 23253 |
| Fam1 | ENSG00000215251 | FASTKD5 | Q7L8L6 | 60493 |
| Fam1 | ENSG00000125871 | MGME1 | Q9BQP7 | 92667 |
| Fam1 | ENSG00000182224 | CYB5D1 | Q6P9G0 | 124637 |
| Fam1 | ENSG00000100296 | THOC5 | Q13769 | 8563 |
| Fam1 | ENSG00000177868 | CCDC23 | Q8N300 | 374969 |
| Fam1 | ENSG00000114544 | SLC41A3 |  | 54946 |
| Fam1 | ENSG00000159685 | CHCHD6 | Q9BRQ6 | 84303 |
| Fam1 | ENSG00000114126 | TFDP2 | Q14188 | 7029 |
| Fam1 | ENSG00000168309 | FAM107A | O95990 | 11170 |
| Fam1 | ENSG00000064042 | LIMCH1 | Q9UPQ0 | 22998 |
| Fam1 | ENSG00000157985 | AGAP1 | Q9UPQ3 | 116987 |
| Fam1 | ENSG00000144908 | ALDH1L1 | O75891 | 10840 |
| Fam1 | ENSG00000183091 | NEB | P20929 | 4703 |
| Fam1 | ENSG00000143369 | ECM1 | Q16610 | 1893 |
| Fam1 | ENSG00000114107 | CEP70 | Q8NHQ1 | 80321 |
| Fam1 | ENSG00000148468 | FAM171A1 | Q5VUB5 | 221061 |
| Fam1 | ENSG00000147687 | TATDN1 |  | 83940 |
| Fam1 | ENSG00000133401 | PDZD2 | O15018 | 23037 |
| Fam1 | ENSG00000178209 | PLEC | Q15149 | 5339 |
| Fam1 | ENSG00000107959 | PITRM1 | Q5JRX3 | 10531 |
| Fam1 | ENSG00000168938 | PPIC | P45877 | 5480 |
| Fam1 | ENSG00000108021 | FAM208B | Q5VWN6 | 54906 |
| Fam1 | ENSG00000057608 | GDI2 | P50395 | 2665 |
| Fam1 | ENSG00000171914 | TLN2 | Q9Y4G6 | 83660 |
| Fam1 | ENSG00000138172 | CALHM2 | Q9HA72 | 51063 |
| Fam1 | ENSG00000241839 | PLEKHO2 | Q8TD55 | 80301 |
| Fam1 | ENSG00000059573 | ALDH18A1 | P54886 | 5832 |
| Fam1 | ENSG00000197321 | SVIL | O95425 | 6840 |
| Fam1 | ENSG00000186628 | FSD2 | A1L4K1 | 123722 |
| Fam1 | ENSG00000171105 | INSR | P06213 | 3643 |
| Fam1 | ENSG00000130164 | LDLR | P01130 | 3949 |
| Fam1 | ENSG00000184076 | UQCR10 | Q9UDW1 | 29796 |
| Fam2 | ENSG00000196352 | CD55 | P08174 | 1604 |
| Fam2 | ENSG00000173706 | HEG1 | Q9ULI3 | 57493 |
| Fam2 | ENSG00000131370 | SH3BP5 | O60239 | 9467 |
| Fam2 | ENSG00000155657 | TTN | Q8WZ42 | 7273 |
| Fam2 | ENSG00000013441 | CLK1 | P49759 | 1195 |
| Fam2 | ENSG00000137962 | ARHGAP29 |  | 9411 |
| Fam2 | ENSG00000179021 | C3orf38 | Q5JPI3 | 285237 |
| Fam2 | ENSG00000113790 | EHHADH | Q08426 | 1962 |
| Fam2 | ENSG00000129071 | MBD4 | O95243 | 8930 |
| Fam2 | ENSG00000084693 | AGBL5 |  | 60509 |
| Fam2 | ENSG00000115275 | MOGS | Q13724 | 7841 |
| Fam2 | ENSG00000134250 | NOTCH2 | Q04721 | 4853 |
| Fam2 | ENSG00000114544 | SLC41A3 |  | 54946 |
| Fam2 | ENSG00000135636 | DYSF | O75923 | 8291 |
| Fam2 | ENSG00000138246 | DNAJC13 | O75165 | 23317 |
| Fam2 | ENSG00000115073 | ACTR1B | P42025 | 10120 |
| Fam2 | ENSG00000143870 | PDIA6 | Q15084 | 10130 |
| Fam2 | ENSG00000135842 | FAM129A |  | 116496 |
| Fam2 | ENSG00000123636 | BAZ2B | Q9UIF8 | 29994 |
| Fam2 | ENSG00000198842 | DUSP27 | Q5VZP5 | 92235 |
| Fam2 | ENSG00000076242 | MLH1 | P40692 | 4292 |
| Fam2 | ENSG00000143164 | DCAF6 | Q58WW2 | 55827 |
| Fam2 | ENSG00000170248 | PDCD6IP | Q8WUM4 | 10015 |
| Fam2 | ENSG00000000971 | CFH | P08603 | 3075 |
| Fam2 | ENSG00000163820 | FYCO1 |  | 79443 |
| Fam2 | ENSG00000116221 | MRPL37 | Q9BZE1 | 51253 |
| Fam2 | ENSG00000114650 | SCAP |  | 22937 |
| Fam2 | ENSG00000204628 | GNB2L1 |  | 619571 |
| Fam2 | ENSG00000145860 | RNF145 | Q96MT1 | 153830 |
| Fam2 | ENSG00000064393 | HIPK2 | Q9H2X6 | 28996 |
| Fam2 | ENSG00000109452 | INPP4B | O15327 | 8821 |
| Fam2 | ENSG00000125347 | IRF1 | P10914 | 3659 |
| Fam2 | ENSG00000069020 | MAST4 |  | 375449 |
| Fam2 | ENSG00000169567 | HINT1 | P49773 | 3094 |
| Fam2 | ENSG00000170469 | SPATA24 |  | 202051 |
| Fam2 | ENSG00000174132 | FAM174A | Q8TBP5 | 345757 |
| Fam2 | ENSG00000145740 | SLC30A5 | Q8TAD4 | 64924 |
| Fam2 | ENSG00000244187 | TMEM141 | Q96I45 | 85014 |
| Fam2 | ENSG00000177239 | MAN1B1 | Q9UKM7 | 11253 |
| Fam2 | ENSG00000134986 | NREP |  | 9315 |
| Fam2 | ENSG00000153130 | SCOC | Q9UIL1 | 60592 |
| Fam2 | ENSG00000107537 | PHYH | O14832 | 5264 |
| Fam2 | ENSG00000105971 | CAV2 | P51636 | 858 |
| Fam2 | ENSG00000185345 | PARK2 | O60260 | 5071 |
| Fam2 | ENSG00000152464 | RPP38 | P78345 | 10557 |
| Fam2 | ENSG00000164292 | RHOBTB3 |  | 22836 |
| Fam2 | ENSG00000113504 | SLC12A7 | Q9Y666 | 10723 |
| Fam2 | ENSG00000137776 | SLTM | Q9NWH9 | 79811 |
| Fam2 | ENSG00000103202 | NME4 |  | 4833 |
| Fam2 | ENSG00000135372 | NAT10 | Q9H0A0 | 55226 |
| Fam2 | ENSG00000171988 | JMJD1C | Q15652 | 221037 |
| Fam2 | ENSG00000110090 | CPT1A | P50416 | 1374 |
| Fam2 | ENSG00000151491 | EPS8 |  | 2059 |
| Fam2 | ENSG00000110011 | DNAJC4 | Q9NNZ3 | 3338 |
| Fam2 | ENSG00000099284 | H2AFY2 | Q9P0M6 | 55506 |
| Fam2 | ENSG00000129493 | HEATR5A |  | 25938 |
| Fam2 | ENSG00000124942 | AHNAK |  | 79026 |
| Fam2 | ENSG00000074527 | NTN4 | Q9HB63 | 59277 |
| Fam2 | ENSG00000135424 | ITGA7 | Q13683 | 3679 |
| Fam2 | ENSG00000155256 | ZFYVE27 | Q5T4F4 | 118813 |
| Fam2 | ENSG00000177981 | ASB8 | Q9H765 | 140461 |
| Fam2 | ENSG00000139116 | KIF21A | Q7Z4S6 | 55605 |
| Fam2 | ENSG00000138119 | MYOF |  | 26509 |
| Fam2 | ENSG00000121316 | PLBD1 | Q6P4A8 | 79887 |
| Fam2 | ENSG00000170430 | MGMT | P16455 | 4255 |
| Fam2 | ENSG00000253797 | UTP14C | Q5TAP6 | 9724 |
| Fam2 | ENSG00000099250 | NRP1 |  | 8829 |
| Fam2 | ENSG00000129925 | TMEM8A | Q9HCN3 | 58986 |
| Fam2 | ENSG00000214530 | STARD10 |  | 10809 |
| Fam2 | ENSG00000065613 | SLK | Q9H2G2 | 9748 |
| Fam2 | ENSG00000100865 | CINP |  | 51550 |
| Fam2 | ENSG00000140983 | RHOT2 | Q8IXI1 | 89941 |
| Fam2 | ENSG00000198131 | ZNF544 |  | 27300 |
| Fam2 | ENSG00000196588 | MKL1 | Q969V6 | 57591 |
| Fam2 | ENSG00000170004 | CHD3 | Q12873 | 1107 |
| Fam2 | ENSG00000141101 | NOB1 | Q9ULX3 | 28987 |
| Fam2 | ENSG00000133026 | MYH10 | P35580 | 4628 |
| Fam2 | ENSG00000066926 | FECH | P22830 | 2235 |
| Fam2 | ENSG00000197879 | MYO1C | O00159 | 4641 |
| Fam2 | ENSG00000103381 | CPPED1 | Q9BRF8 | 55313 |
| Fam2 | ENSG00000198947 | DMD | P11532 | 1756 |
| Fam2 | ENSG00000167705 | RILP |  | 83547 |
| Fam2 | ENSG00000141219 | C17orf80 |  | 55028 |
| Fam2 | ENSG00000130733 | YIPF2 | Q9BWQ6 | 78992 |
| Fam2 | ENSG00000134765 | DSC1 | Q08554 | 1823 |
| Fam2 | ENSG00000100271 | TTLL1 | O95922 | 25809 |
| Fam2 | ENSG00000141540 | TTYH2 | Q9BSA4 | 94015 |
| Fam2 | ENSG00000185437 | SH3BGR | P55822 | 6450 |
| Fam2 | ENSG00000161649 | CD300LG | Q6UXG3 | 146894 |
| Fam2 | ENSG00000076662 | ICAM3 | P32942 | 3385 |
| Fam2 | ENSG00000166747 | AP1G1 | O43747 | 164 |
| Fam2 | ENSG00000006744 | ELAC2 | Q9BQ52 | 60528 |
| Fam2 | ENSG00000142156 | COL6A1 | P12109 | 1291 |
| Fam2 | ENSG00000070366 | SMG6 | Q86US8 | 23293 |
| Fam2 | ENSG00000132359 | RAP1GAP2 | Q684P5 | 23108 |
| Fam2 | ENSG00000147113 | CXorf36 | Q9H7Y0 | 79742 |
| Fam2 | ENSG00000089199 | CHGB | P05060 | 1114 |
| Fam2 | ENSG00000163092 | XIRP2 | A4UGR9 | 129446 |
| Fam2 | ENSG00000082781 | ITGB5 | P18084 | 3693 |
| Fam2 | ENSG00000011566 | MAP4K3 | Q8IVH8 | 8491 |
| Fam2 | ENSG00000162923 | WDR26 | Q9H7D7 | 80232 |
| Fam2 | ENSG00000121897 | LIAS | O43766 | 11019 |
| Fam2 | ENSG00000170275 | CRTAP | O75718 | 100653071 |
| Fam2 | ENSG00000114867 | EIF4G1 | Q04637 | 1981 |
| Fam2 | ENSG00000077097 | TOP2B |  | 7155 |
| Fam2 | ENSG00000154358 | OBSCN | Q5VST9 | 84033 |
| Fam2 | ENSG00000168297 | PXK | Q7Z7A4 | 54899 |
| Fam2 | ENSG00000115457 | IGFBP2 |  | 3485 |
| Fam2 | ENSG00000198399 | ITSN2 | Q9NZM3 | 50618 |
| Fam2 | ENSG00000162645 | GBP2 | P32456 | 2634 |
| Fam2 | ENSG00000168924 | LETM1 | O95202 | 3954 |
| Fam2 | ENSG00000114054 | PCCB | P05166 | 5096 |
| Fam2 | ENSG00000154222 | CC2D1B | Q5T0F9 | 200014 |
| Fam2 | ENSG00000178104 | PDE4DIP | Q5VU43 | 101930416 |
| Fam2 | ENSG00000136536 | MARCH7 | Q9H992 | 64844 |
| Fam2 | ENSG00000116991 | SIPA1L2 | Q9P2F8 | 57568 |
| Fam2 | ENSG00000134253 | TRIM45 |  | 80263 |
| Fam2 | ENSG00000135744 | AGT | P01019 | 183 |
| Fam2 | ENSG00000115129 | TP53I3 | Q53FA7 | 9540 |
| Fam2 | ENSG00000143891 | GALM | Q96C23 | 130589 |
| Fam2 | ENSG00000162688 | AGL | P35573 | 178 |
| Fam2 | ENSG00000064225 | ST3GAL6 | Q9Y274 | 10402 |
| Fam2 | ENSG00000144908 | ALDH1L1 | O75891 | 10840 |
| Fam2 | ENSG00000087274 | ADD1 |  | 118 |
| Fam2 | ENSG00000183873 | SCN5A |  | 6331 |
| Fam2 | ENSG00000162909 | CAPN2 |  | 824 |
| Fam2 | ENSG00000134313 | KIDINS220 |  | 57498 |
| Fam2 | ENSG00000121440 | PDZRN3 |  | 23024 |
| Fam2 | ENSG00000116729 | WLS | Q5T9L3 | 79971 |
| Fam2 | ENSG00000163131 | CTSS | P25774 | 1520 |
| Fam2 | ENSG00000138031 | ADCY3 | O60266 | 109 |
| Fam2 | ENSG00000114107 | CEP70 | Q8NHQ1 | 80321 |
| Fam2 | ENSG00000221818 | EBF2 | Q9HAK2 | 64641 |
| Fam2 | ENSG00000185963 | BICD2 | Q8TD16 | 23299 |
| Fam2 | ENSG00000151914 | DST | Q03001 | 667 |
| Fam2 | ENSG00000036448 | MYOM2 | P54296 | 9172 |
| Fam2 | ENSG00000106524 | ANKMY2 | Q8IV38 | 57037 |
| Fam2 | ENSG00000106460 | TMEM106B | Q9NUM4 | 54664 |
| Fam2 | ENSG00000107959 | PITRM1 | Q5JRX3 | 10531 |
| Fam2 | ENSG00000197969 | VPS13A | Q96RL7 | 23230 |
| Fam2 | ENSG00000070061 | IKBKAP |  | 8518 |
| Fam2 | ENSG00000119328 | FAM206A | Q9NX38 | 54942 |
| Fam2 | ENSG00000172159 | FRMD3 | A2A2Y4 | 257019 |
| Fam2 | ENSG00000083857 | FAT1 | Q14517 | 2195 |
| Fam2 | ENSG00000130348 | QRSL1 | Q9H0R6 | 55278 |
| Fam2 | ENSG00000158863 | FAM160B2 |  | 64760 |
| Fam2 | ENSG00000124523 | SIRT5 |  | 23408 |
| Fam2 | ENSG00000148468 | FAM171A1 | Q5VUB5 | 221061 |
| Fam2 | ENSG00000128563 | PRKRIP1 | Q9H875 | 100630923 |
| Fam2 | ENSG00000198876 | DCAF12 | Q5T6F0 | 25853 |
| Fam2 | ENSG00000154079 | C6orf57 | Q5VUM1 | 135154 |
| Fam2 | ENSG00000095380 | NANS | Q9NR45 | 54187 |
| Fam2 | ENSG00000117868 | ESYT2 | A0FGR8 | 57488 |
| Fam2 | ENSG00000049540 | ELN |  | 2006 |
| Fam2 | ENSG00000241644 | INMT | O95050 | 11185 |
| Fam2 | ENSG00000113360 | DROSHA | Q9NRR4 | 29102 |
| Fam2 | ENSG00000188186 | LAMTOR4 | Q0VGL1 | 389541 |
| Fam2 | ENSG00000104635 | SLC39A14 | Q15043 | 23516 |
| Fam2 | ENSG00000132953 | XPO4 | Q9C0E2 | 64328 |
| Fam2 | ENSG00000100938 | GMPR2 | Q9P2T1 | 51292 |
| Fam2 | ENSG00000136111 | TBC1D4 | O60343 | 9882 |
| Fam2 | ENSG00000174744 | BRMS1 |  | 25855 |
| Fam2 | ENSG00000119682 | AREL1 |  | 9870 |
| Fam2 | ENSG00000127329 | PTPRB | P23467 | 5787 |
| Fam2 | ENSG00000136153 | LMO7 | Q8WWI1 | 4008 |
| Fam2 | ENSG00000175198 | PCCA | P05165 | 5095 |
| Fam2 | ENSG00000162222 | TTC9C |  | 283237 |
| Fam2 | ENSG00000054654 | SYNE2 | Q8WXH0 | 23224 |
| Fam2 | ENSG00000182667 | NTM | Q9P121 | 50863 |
| Fam2 | ENSG00000150527 | CTAGE5 |  | 4253 |
| Fam2 | ENSG00000179364 | PACS2 |  | 101929695 |
| Fam2 | ENSG00000247077 | PGAM5 | Q96HS1 | 192111 |
| Fam2 | ENSG00000028203 | VEZT | Q9HBM0 | 55591 |
| Fam2 | ENSG00000140553 | UNC45A | Q9H3U1 | 55898 |
| Fam2 | ENSG00000119681 | LTBP2 | Q14767 | 4053 |
| Fam2 | ENSG00000198431 | TXNRD1 | Q16881 | 7296 |
| Fam2 | ENSG00000172890 | NADSYN1 | Q6IA69 | 55191 |
| Fam2 | ENSG00000176915 | ANKLE2 |  | 23141 |
| Fam2 | ENSG00000204842 | ATXN2 |  | 6311 |
| Fam2 | ENSG00000102805 | CLN5 | O75503 | 1203 |
| Fam2 | ENSG00000151320 | AKAP6 | Q13023 | 9472 |
| Fam2 | ENSG00000182979 | MTA1 | Q13330 | 9112 |
| Fam2 | ENSG00000177354 | C10orf71 | Q711Q0 | 118461 |
| Fam2 | ENSG00000133103 | COG6 | Q9Y2V7 | 57511 |
| Fam2 | ENSG00000119906 | FAM178A | Q8IX21 | 55719 |
| Fam2 | ENSG00000155229 | MMS19 |  | 64210 |
| Fam2 | ENSG00000120690 | ELF1 | P32519 | 1997 |
| Fam2 | ENSG00000136152 | COG3 | Q96JB2 | 83548 |
| Fam2 | ENSG00000100359 | SGSM3 |  | 27352 |
| Fam2 | ENSG00000100345 | MYH9 | P35579 | 4627 |
| Fam2 | ENSG00000103051 | COG4 |  | 25839 |
| Fam2 | ENSG00000054611 | TBC1D22A | Q8WUA7 | 25771 |
| Fam2 | ENSG00000125863 | MKKS | Q9NPJ1 | 8195 |
| Fam2 | ENSG00000124198 | ARFGEF2 | Q9Y6D5 | 10564 |
| Fam2 | ENSG00000141959 | PFKL | P17858 | 5211 |
| Fam2 | ENSG00000088367 | EPB41L1 | Q9H4G0 | 2036 |
| Fam2 | ENSG00000169084 | DHRSX | Q8N5I4 | 207063 |
| Fam2 | ENSG00000130164 | LDLR | P01130 | 3949 |
| Fam2 | ENSG00000158470 | B4GALT5 | O43286 | 9334 |
| Fam2 | ENSG00000183255 | PTTG1IP |  | 754 |
| Fam2 | ENSG00000179776 | CDH5 | P33151 | 1003 |
| Fam2 | ENSG00000104774 | MAN2B1 | O00754 | 4125 |
| Fam2 | ENSG00000128335 | APOL2 | Q9BQE5 | 23780 |
| Fam2 | ENSG00000167393 | PPP2R3B | Q9Y5P8 | 28227 |
| Fam2 | ENSG00000140740 | UQCRC2 | P22695 | 7385 |
| Fam2 | ENSG00000101439 | CST3 | P01034 | 1471 |
| Fam2 | ENSG00000132670 | PTPRA | P18433 | 5786 |
| Fam2 | ENSG00000129347 | KRI1 | Q8N9T8 | 65095 |
| Fam2 | ENSG00000092929 | UNC13D |  | 201294 |
| Fam2 | ENSG00000103342 | GSPT1 | P15170 | 2935 |
| Fam2 | ENSG00000008710 | PKD1 | P98161 | 101930075 |
| Fam2 | ENSG00000141052 | MYOCD |  | 93649 |

**Supplementary Table S2**

| **Ensg** | **INFO** |
| --- | --- |
| ENSG00000003756 | SOURCE=GO.BP; GO.term.name=negative regulation of cell proliferation; GO.term.ID=GO:0008285; Evidence.code=TAS; Information.content=3.53 |
| ENSG00000004975 | SOURCE=GO.BP; GO.term.name=heart development; GO.term.ID=GO:0007507; Evidence.code=ISS; Information.content=3.25 |
| ENSG00000008710 | SOURCE=GO.BP; GO.term.name=heart development; GO.term.ID=GO:0007507; Evidence.code=IEP; Information.content=3.25 |
| ENSG00000015479 | SOURCE=PPI.MENTHA; SCORE=0.236; PMID=26496610 |
| ENSG00000018625 | SOURCE=GO.BP; GO.term.name=cell communication by electrical coupling involved in cardiac conduction; GO.term.ID=GO:0086064; Evidence.code=TAS; Information.content=5.53 |
| ENSG00000025434 | SOURCE=GO.BP; GO.term.name=lipid homeostasis; GO.term.ID=GO:0055088; Evidence.code=ISS; Information.content=3.95 |
| ENSG00000027075 | SOURCE=GO.BP; GO.term.name=regulation of bicellular tight junction assembly; GO.term.ID=GO:2000810; Evidence.code=IMP; Information.content=5.33 |
| ENSG00000030582 | SOURCE=COMPLEX.BIOPLEX; SCORE=0.997709132; ORG_UNIPROT=2896; SOURCE=PPI.MENTHA; SCORE=0.126; PMID=26186194 |
| ENSG00000035403 | SOURCE=GO.BP; GO.term.name=negative regulation of cell migration; GO.term.ID=GO:0030336; Evidence.code=TAS; Information.content=4.04 |
| ENSG00000036257 | SOURCE=PPI.MENTHA; SCORE=0.126; PMID=21145461 |
| ENSG00000046604 | SOURCE=PPI.STRING; SCORE=967; ORG_ENSP=ENSP00000261590; SOURCE=PPI.MENTHA; SCORE=0.21; PMID=11790773 ; SOURCE=GO.BP; GO.term.name=regulation of heart rate by cardiac conduction; GO.term.ID=GO:0086091; Evidence.code=IMP; Information.content=5 |
| ENSG00000048052 | SOURCE=GO.BP; GO.term.name=heart development; GO.term.ID=GO:0007507; Evidence.code=ISS; Information.content=3.25 |
| ENSG00000048140 | SOURCE=GO.BP; GO.term.name=establishment of protein localization to plasma membrane; GO.term.ID=GO:0090002; Evidence.code=IBA; Information.content=4.08 |
| ENSG00000053918 | SOURCE=GO.BP; GO.term.name=regulation of heart rate by cardiac conduction; GO.term.ID=GO:0086091; Evidence.code=IMP; Information.content=5 |
| ENSG00000055118 | SOURCE=GO.BP; GO.term.name=regulation of heart rate by cardiac conduction; GO.term.ID=GO:0086091; Evidence.code=IMP; Information.content=5 |
| ENSG00000055130 | SOURCE=GO.BP; GO.term.name=negative regulation of cell proliferation; GO.term.ID=GO:0008285; Evidence.code=TAS; Information.content=3.53 |
| ENSG00000055208 | SOURCE=GO.BP; GO.term.name=heart development; GO.term.ID=GO:0007507; Evidence.code=IMP; Information.content=3.25 |
| ENSG00000057294 | SOURCE=Ens86Paralog; IDENTITY=22 |
| ENSG00000067113 | SOURCE=GO.BP; GO.term.name=negative regulation of cell proliferation; GO.term.ID=GO:0008285; Evidence.code=NAS; Information.content=3.53 |
| ENSG00000067182 | SOURCE=GO.BP; GO.term.name=establishment of protein localization to plasma membrane; GO.term.ID=GO:0090002; Evidence.code=IMP; Information.content=4.08 |
| ENSG00000067900 | SOURCE=GO.BP; GO.term.name=establishment of protein localization to plasma membrane; GO.term.ID=GO:0090002; Evidence.code=IGI; Information.content=4.08; SOURCE=GO.BP; GO.term.name=establishment of protein localization to plasma membrane; GO.term.ID=GO:0090002; Evidence.code=IMP; Information.content=4.08 |
| ENSG00000068305 | SOURCE=GO.BP; GO.term.name=heart development; GO.term.ID=GO:0007507; Evidence.code=IEP; Information.content=3.25 |
| ENSG00000068903 | SOURCE=GO.BP; GO.term.name=negative regulation of cell proliferation; GO.term.ID=GO:0008285; Evidence.code=IMP; Information.content=3.53 |
| ENSG00000071051 | SOURCE=GO.BP; GO.term.name=negative regulation of cell proliferation; GO.term.ID=GO:0008285; Evidence.code=TAS; Information.content=3.53 |
| ENSG00000071242 | SOURCE=GO.BP; GO.term.name=negative regulation of cell proliferation; GO.term.ID=GO:0008285; Evidence.code=IDA; Information.content=3.53 |
| ENSG00000072062 | SOURCE=GO.BP; GO.term.name=cell communication by electrical coupling involved in cardiac conduction; GO.term.ID=GO:0086064; Evidence.code=TAS; Information.content=5.53; SOURCE=GO.BP; GO.term.name=regulation of bicellular tight junction assembly; GO.term.ID=GO:2000810; Evidence.code=IDA; Information.content=5.33 |
| ENSG00000072195 | SOURCE=GO.BP; GO.term.name=negative regulation of cell proliferation; GO.term.ID=GO:0008285; Evidence.code=TAS; Information.content=3.53 |
| ENSG00000072778 | SOURCE=GO.BP; GO.term.name=lipid homeostasis; GO.term.ID=GO:0055088; Evidence.code=IBA; Information.content=3.95 |
| ENSG00000075142 | SOURCE=GO.BP; GO.term.name=heart development; GO.term.ID=GO:0007507; Evidence.code=TAS; Information.content=3.25 |
| ENSG00000075413 | SOURCE=PPI.STRING; SCORE=929; ORG_ENSP=ENSP00000411397; SOURCE=PPI.MENTHA; SCORE=0.659; PMID=12941695 |
| ENSG00000075711 | SOURCE=PPI.STRING; SCORE=834; ORG_ENSP=ENSP00000345731 |
| ENSG00000077522 | SOURCE=GO.BP; GO.term.name=establishment of protein localization to plasma membrane; GO.term.ID=GO:0090002; Evidence.code=IMP; Information.content=4.08 |
| ENSG00000078304 | SOURCE=GO.BP; GO.term.name=negative regulation of cell proliferation; GO.term.ID=GO:0008285; Evidence.code=IDA; Information.content=3.53 |
| ENSG00000080503 | SOURCE=GO.BP; GO.term.name=negative regulation of cell proliferation; GO.term.ID=GO:0008285; Evidence.code=IDA; Information.content=3.53 |
| ENSG00000081189 | SOURCE=GO.BP; GO.term.name=heart development; GO.term.ID=GO:0007507; Evidence.code=IEP; Information.content=3.25; SOURCE=GO.BP; GO.term.name=heart development; GO.term.ID=GO:0007507; Evidence.code=ISS; Information.content=3.25; SOURCE=GO.BP; GO.term.name=heart development; GO.term.ID=GO:0007507; Evidence.code=NAS; Information.content=3.25 |
| ENSG00000085872 | SOURCE=GO.BP; GO.term.name=negative regulation of cell proliferation; GO.term.ID=GO:0008285; Evidence.code=IDA; Information.content=3.53 |
| ENSG00000087008 | SOURCE=GO.BP; GO.term.name=lipid homeostasis; GO.term.ID=GO:0055088; Evidence.code=IBA; Information.content=3.95 |
| ENSG00000088992 | SOURCE=GO.BP; GO.term.name=negative regulation of cell proliferation; GO.term.ID=GO:0008285; Evidence.code=IMP; Information.content=3.53 |
| ENSG00000089225 | SOURCE=GO.BP; GO.term.name=heart development; GO.term.ID=GO:0007507; Evidence.code=IDA; Information.content=3.25; SOURCE=GO.BP; GO.term.name=heart development; GO.term.ID=GO:0007507; Evidence.code=IMP; Information.content=3.25; SOURCE=GO.BP; GO.term.name=negative regulation of cell proliferation; GO.term.ID=GO:0008285; Evidence.code=IDA; Information.content=3.53; SOURCE=GO.BP; GO.term.name=negative regulation of cell migration; GO.term.ID=GO:0030336; Evidence.code=IDA; Information.content=4.04 |
| ENSG00000092820 | SOURCE=GO.BP; GO.term.name=establishment of protein localization to plasma membrane; GO.term.ID=GO:0090002; Evidence.code=IMP; Information.content=4.08 |
| ENSG00000096696 | SOURCE=PPI.STRING; SCORE=984; ORG_ENSP=ENSP00000369129; SOURCE=PPI.MENTHA; SCORE=0.659; PMID=25225338 11790773 ; SOURCE=GO.BP; GO.term.name=regulation of heart rate by cardiac conduction; GO.term.ID=GO:0086091; Evidence.code=IMP; Information.content=5 |
| ENSG00000096968 | SOURCE=GO.BP; GO.term.name=negative regulation of cell proliferation; GO.term.ID=GO:0008285; Evidence.code=ISS; Information.content=3.53 |
| ENSG00000099956 | SOURCE=GO.BP; GO.term.name=negative regulation of cell proliferation; GO.term.ID=GO:0008285; Evidence.code=IBA; Information.content=3.53 |
| ENSG00000100311 | SOURCE=GO.BP; GO.term.name=heart development; GO.term.ID=GO:0007507; Evidence.code=ISS; Information.content=3.25 |
| ENSG00000100714 | SOURCE=GO.BP; GO.term.name=heart development; GO.term.ID=GO:0007507; Evidence.code=ISS; Information.content=3.25 |
| ENSG00000100852 | SOURCE=PPI.STRING; SCORE=702; ORG_ENSP=ENSP00000371897 |
| ENSG00000101665 | SOURCE=GO.BP; GO.term.name=negative regulation of cell migration; GO.term.ID=GO:0030336; Evidence.code=TAS; Information.content=4.04 |
| ENSG00000102125 | SOURCE=GO.BP; GO.term.name=heart development; GO.term.ID=GO:0007507; Evidence.code=IMP; Information.content=3.25 |
| ENSG00000102572 | SOURCE=GO.BP; GO.term.name=negative regulation of cell migration; GO.term.ID=GO:0030336; Evidence.code=IMP; Information.content=4.04 |
| ENSG00000102760 | SOURCE=GO.BP; GO.term.name=negative regulation of cell proliferation; GO.term.ID=GO:0008285; Evidence.code=IMP; Information.content=3.53 |
| ENSG00000102974 | SOURCE=Ensembl,Oreganno |
| ENSG00000103197 | SOURCE=GO.BP; GO.term.name=heart development; GO.term.ID=GO:0007507; Evidence.code=ISS; Information.content=3.25; SOURCE=GO.BP; GO.term.name=negative regulation of cell proliferation; GO.term.ID=GO:0008285; Evidence.code=ISS; Information.content=3.53 |
| ENSG00000103423 | SOURCE=GO.BP; GO.term.name=negative regulation of cell proliferation; GO.term.ID=GO:0008285; Evidence.code=IDA; Information.content=3.53 |
| ENSG00000104332 | SOURCE=GO.BP; GO.term.name=negative regulation of cell proliferation; GO.term.ID=GO:0008285; Evidence.code=IDA; Information.content=3.53; SOURCE=GO.BP; GO.term.name=negative regulation of cell migration; GO.term.ID=GO:0030336; Evidence.code=IDA; Information.content=4.04 |
| ENSG00000105185 | SOURCE=GO.BP; GO.term.name=negative regulation of cell proliferation; GO.term.ID=GO:0008285; Evidence.code=IMP; Information.content=3.53 |
| ENSG00000105329 | SOURCE=GO.BP; GO.term.name=negative regulation of cell proliferation; GO.term.ID=GO:0008285; Evidence.code=IDA; Information.content=3.53 |
| ENSG00000105409 | SOURCE=GO.BP; GO.term.name=cell communication by electrical coupling involved in cardiac conduction; GO.term.ID=GO:0086064; Evidence.code=TAS; Information.content=5.53 |
| ENSG00000105607 | SOURCE=GO.BP; GO.term.name=lipid homeostasis; GO.term.ID=GO:0055088; Evidence.code=IBA; Information.content=3.95 |
| ENSG00000105698 | SOURCE=GO.BP; GO.term.name=lipid homeostasis; GO.term.ID=GO:0055088; Evidence.code=ISS; Information.content=3.95 |
| ENSG00000105711 | SOURCE=GO.BP; GO.term.name=positive regulation of sodium ion transport; GO.term.ID=GO:0010765; Evidence.code=IDA; Information.content=4.9; SOURCE=GO.BP; GO.term.name=regulation of heart rate by cardiac conduction; GO.term.ID=GO:0086091; Evidence.code=IMP; Information.content=5 |
| ENSG00000105928 | SOURCE=GO.BP; GO.term.name=negative regulation of cell proliferation; GO.term.ID=GO:0008285; Evidence.code=IDA; Information.content=3.53 |
| ENSG00000105974 | SOURCE=GO.BP; GO.term.name=regulation of heart rate by cardiac conduction; GO.term.ID=GO:0086091; Evidence.code=ISS; Information.content=5 |
| ENSG00000106290 | SOURCE=GO.BP; GO.term.name=negative regulation of cell proliferation; GO.term.ID=GO:0008285; Evidence.code=IDA; Information.content=3.53 |
| ENSG00000106483 | SOURCE=GO.BP; GO.term.name=negative regulation of cell proliferation; GO.term.ID=GO:0008285; Evidence.code=IMP; Information.content=3.53 |
| ENSG00000106991 | SOURCE=GO.BP; GO.term.name=negative regulation of cell migration; GO.term.ID=GO:0030336; Evidence.code=IDA; Information.content=4.04 |
| ENSG00000107104 | SOURCE=GO.BP; GO.term.name=negative regulation of cell migration; GO.term.ID=GO:0030336; Evidence.code=IMP; Information.content=4.04 |
| ENSG00000107937 | SOURCE=GO.BP; GO.term.name=negative regulation of cell proliferation; GO.term.ID=GO:0008285; Evidence.code=IMP; Information.content=3.53; SOURCE=GO.BP; GO.term.name=negative regulation of cell migration; GO.term.ID=GO:0030336; Evidence.code=IDA; Information.content=4.04 |
| ENSG00000108094 | SOURCE=GO.BP; GO.term.name=negative regulation of cell proliferation; GO.term.ID=GO:0008285; Evidence.code=TAS; Information.content=3.53 |
| ENSG00000108219 | SOURCE=GO.BP; GO.term.name=establishment of protein localization to plasma membrane; GO.term.ID=GO:0090002; Evidence.code=IMP; Information.content=4.08 |
| ENSG00000108953 | SOURCE=PPI.MENTHA; SCORE=0.126; PMID=17979178 ; SOURCE=GO.BP; GO.term.name=regulation of heart rate by cardiac conduction; GO.term.ID=GO:0086091; Evidence.code=IC; Information.content=5 |
| ENSG00000109062 | SOURCE=GO.BP; GO.term.name=negative regulation of cell proliferation; GO.term.ID=GO:0008285; Evidence.code=IDA; Information.content=3.53; SOURCE=GO.BP; GO.term.name=negative regulation of cell proliferation; GO.term.ID=GO:0008285; Evidence.code=IMP; Information.content=3.53; SOURCE=GO.BP; GO.term.name=establishment of protein localization to plasma membrane; GO.term.ID=GO:0090002; Evidence.code=IMP; Information.content=4.08 |
| ENSG00000109670 | SOURCE=GO.BP; GO.term.name=lipid homeostasis; GO.term.ID=GO:0055088; Evidence.code=ISS; Information.content=3.95 |
| ENSG00000109756 | SOURCE=GO.BP; GO.term.name=negative regulation of cell proliferation; GO.term.ID=GO:0008285; Evidence.code=IDA; Information.content=3.53 |
| ENSG00000111077 | SOURCE=GO.BP; GO.term.name=negative regulation of cell proliferation; GO.term.ID=GO:0008285; Evidence.code=IMP; Information.content=3.53 |
| ENSG00000111245 | SOURCE=GO.BP; GO.term.name=heart development; GO.term.ID=GO:0007507; Evidence.code=ISS; Information.content=3.25 |
| ENSG00000111271 | SOURCE=GO.BP; GO.term.name=lipid homeostasis; GO.term.ID=GO:0055088; Evidence.code=IBA; Information.content=3.95 |
| ENSG00000111276 | SOURCE=GO.BP; GO.term.name=negative regulation of cell proliferation; GO.term.ID=GO:0008285; Evidence.code=IDA; Information.content=3.53; SOURCE=GO.BP; GO.term.name=negative regulation of cell proliferation; GO.term.ID=GO:0008285; Evidence.code=IMP; Information.content=3.53 |
| ENSG00000111653 | SOURCE=GO.BP; GO.term.name=negative regulation of cell proliferation; GO.term.ID=GO:0008285; Evidence.code=IDA; Information.content=3.53 |
| ENSG00000112096 | SOURCE=GO.BP; GO.term.name=negative regulation of cell proliferation; GO.term.ID=GO:0008285; Evidence.code=IMP; Information.content=3.53 |
| ENSG00000112276 | SOURCE=GO.BP; GO.term.name=heart development; GO.term.ID=GO:0007507; Evidence.code=IMP; Information.content=3.25 |
| ENSG00000112658 | SOURCE=GO.BP; GO.term.name=heart development; GO.term.ID=GO:0007507; Evidence.code=ISS; Information.content=3.25 |
| ENSG00000113360 | SOURCE=PPI.MENTHA; SCORE=0.126; PMID=24778252 |
| ENSG00000113657 | SOURCE=GO.BP; GO.term.name=negative regulation of cell migration; GO.term.ID=GO:0030336; Evidence.code=ISS; Information.content=4.04 |
| ENSG00000114166 | SOURCE=GO.BP; GO.term.name=negative regulation of cell proliferation; GO.term.ID=GO:0008285; Evidence.code=IDA; Information.content=3.53 |
| ENSG00000114279 | SOURCE=GO.BP; GO.term.name=heart development; GO.term.ID=GO:0007507; Evidence.code=TAS; Information.content=3.25 |
| ENSG00000115415 | SOURCE=Oreganno |
| ENSG00000115461 | SOURCE=GO.BP; GO.term.name=negative regulation of cell migration; GO.term.ID=GO:0030336; Evidence.code=IDA; Information.content=4.04 |
| ENSG00000115808 | SOURCE=GO.BP; GO.term.name=negative regulation of cell proliferation; GO.term.ID=GO:0008285; Evidence.code=IMP; Information.content=3.53 |
| ENSG00000116016 | SOURCE=Oreganno,TFe |
| ENSG00000117054 | SOURCE=GO.BP; GO.term.name=lipid homeostasis; GO.term.ID=GO:0055088; Evidence.code=IBA; Information.content=3.95 |
| ENSG00000117298 | SOURCE=GO.BP; GO.term.name=heart development; GO.term.ID=GO:0007507; Evidence.code=IMP; Information.content=3.25 |
| ENSG00000117595 | SOURCE=GO.BP; GO.term.name=negative regulation of cell proliferation; GO.term.ID=GO:0008285; Evidence.code=IDA; Information.content=3.53 |
| ENSG00000117707 | SOURCE=GO.BP; GO.term.name=negative regulation of cell proliferation; GO.term.ID=GO:0008285; Evidence.code=IMP; Information.content=3.53 |
| ENSG00000118046 | SOURCE=GO.BP; GO.term.name=negative regulation of cell proliferation; GO.term.ID=GO:0008285; Evidence.code=IMP; Information.content=3.53 |
| ENSG00000118515 | SOURCE=GO.BP; GO.term.name=positive regulation of sodium ion transport; GO.term.ID=GO:0010765; Evidence.code=IBA; Information.content=4.9 |
| ENSG00000118762 | SOURCE=GO.BP; GO.term.name=heart development; GO.term.ID=GO:0007507; Evidence.code=IEP; Information.content=3.25; SOURCE=GO.BP; GO.term.name=negative regulation of cell proliferation; GO.term.ID=GO:0008285; Evidence.code=NAS; Information.content=3.53 |
| ENSG00000119185 | SOURCE=GO.BP; GO.term.name=negative regulation of cell proliferation; GO.term.ID=GO:0008285; Evidence.code=IDA; Information.content=3.53 |
| ENSG00000119699 | SOURCE=GO.BP; GO.term.name=negative regulation of cell proliferation; GO.term.ID=GO:0008285; Evidence.code=ISS; Information.content=3.53 |
| ENSG00000119917 | SOURCE=GO.BP; GO.term.name=negative regulation of cell proliferation; GO.term.ID=GO:0008285; Evidence.code=IDA; Information.content=3.53 |
| ENSG00000119950 | SOURCE=GO.BP; GO.term.name=negative regulation of cell proliferation; GO.term.ID=GO:0008285; Evidence.code=TAS; Information.content=3.53 |
| ENSG00000120156 | SOURCE=GO.BP; GO.term.name=heart development; GO.term.ID=GO:0007507; Evidence.code=ISS; Information.content=3.25 |
| ENSG00000120318 | SOURCE=GO.BP; GO.term.name=negative regulation of cell migration; GO.term.ID=GO:0030336; Evidence.code=ISS; Information.content=4.04 |
| ENSG00000120738 | SOURCE=Oreganno |
| ENSG00000121769 | SOURCE=GO.BP; GO.term.name=negative regulation of cell proliferation; GO.term.ID=GO:0008285; Evidence.code=TAS; Information.content=3.53 |
| ENSG00000122591 | SOURCE=GO.BP; GO.term.name=establishment of protein localization to plasma membrane; GO.term.ID=GO:0090002; Evidence.code=IDA; Information.content=4.08 |
| ENSG00000122971 | SOURCE=GO.BP; GO.term.name=lipid homeostasis; GO.term.ID=GO:0055088; Evidence.code=IBA; Information.content=3.95 |
| ENSG00000123080 | SOURCE=GO.BP; GO.term.name=negative regulation of cell proliferation; GO.term.ID=GO:0008285; Evidence.code=IDA; Information.content=3.53 |
| ENSG00000123728 | SOURCE=GO.BP; GO.term.name=negative regulation of cell migration; GO.term.ID=GO:0030336; Evidence.code=ISS; Information.content=4.04 |
| ENSG00000123933 | SOURCE=GO.BP; GO.term.name=negative regulation of cell proliferation; GO.term.ID=GO:0008285; Evidence.code=TAS; Information.content=3.53 |
| ENSG00000124762 | SOURCE=GO.BP; GO.term.name=negative regulation of cell proliferation; GO.term.ID=GO:0008285; Evidence.code=IDA; Information.content=3.53; SOURCE=GO.BP; GO.term.name=negative regulation of cell proliferation; GO.term.ID=GO:0008285; Evidence.code=IMP; Information.content=3.53 |
| ENSG00000124802 | SOURCE=GO.BP; GO.term.name=negative regulation of cell proliferation; GO.term.ID=GO:0008285; Evidence.code=ISS; Information.content=3.53 |
| ENSG00000125266 | SOURCE=COMPLEX.BIOPLEX; SCORE=0.93141141; ORG_UNIPROT=1948; SOURCE=PPI.MENTHA; SCORE=0.126; PMID=26186194 |
| ENSG00000125347 | SOURCE=GO.BP; GO.term.name=negative regulation of cell proliferation; GO.term.ID=GO:0008285; Evidence.code=TAS; Information.content=3.53 |
| ENSG00000125378 | SOURCE=GO.BP; GO.term.name=negative regulation of cell proliferation; GO.term.ID=GO:0008285; Evidence.code=IDA; Information.content=3.53 |
| ENSG00000125863 | SOURCE=GO.BP; GO.term.name=heart development; GO.term.ID=GO:0007507; Evidence.code=TAS; Information.content=3.25 |
| ENSG00000126062 | SOURCE=GO.BP; GO.term.name=negative regulation of cell proliferation; GO.term.ID=GO:0008285; Evidence.code=NAS; Information.content=3.53 |
| ENSG00000127863 | SOURCE=COMPLEX.BIOPLEX; SCORE=0.774392908; ORG_UNIPROT=55504 |
| ENSG00000128245 | SOURCE=PPI.MENTHA; SCORE=0.126; PMID=17979178 |
| ENSG00000128917 | SOURCE=GO.BP; GO.term.name=negative regulation of cell proliferation; GO.term.ID=GO:0008285; Evidence.code=IMP; Information.content=3.53 |
| ENSG00000128918 | SOURCE=GO.BP; GO.term.name=negative regulation of cell proliferation; GO.term.ID=GO:0008285; Evidence.code=IDA; Information.content=3.53 |
| ENSG00000128928 | SOURCE=GO.BP; GO.term.name=lipid homeostasis; GO.term.ID=GO:0055088; Evidence.code=IBA; Information.content=3.95 |
| ENSG00000129355 | SOURCE=GO.BP; GO.term.name=negative regulation of cell proliferation; GO.term.ID=GO:0008285; Evidence.code=IDA; Information.content=3.53 |
| ENSG00000129991 | SOURCE=GO.BP; GO.term.name=heart development; GO.term.ID=GO:0007507; Evidence.code=ISS; Information.content=3.25 |
| ENSG00000130037 | SOURCE=GO.BP; GO.term.name=regulation of heart rate by cardiac conduction; GO.term.ID=GO:0086091; Evidence.code=IMP; Information.content=5 |
| ENSG00000130147 | SOURCE=GO.BP; GO.term.name=negative regulation of cell proliferation; GO.term.ID=GO:0008285; Evidence.code=IMP; Information.content=3.53 |
| ENSG00000130303 | SOURCE=GO.BP; GO.term.name=negative regulation of cell migration; GO.term.ID=GO:0030336; Evidence.code=IDA; Information.content=4.04 |
| ENSG00000131477 | SOURCE=GO.BP; GO.term.name=heart development; GO.term.ID=GO:0007507; Evidence.code=ISS; Information.content=3.25 |
| ENSG00000132155 | SOURCE=GO.BP; GO.term.name=negative regulation of cell proliferation; GO.term.ID=GO:0008285; Evidence.code=IDA; Information.content=3.53 |
| ENSG00000132294 | SOURCE=GO.BP; GO.term.name=establishment of protein localization to plasma membrane; GO.term.ID=GO:0090002; Evidence.code=IMP; Information.content=4.08 |
| ENSG00000132589 | SOURCE=GO.BP; GO.term.name=establishment of protein localization to plasma membrane; GO.term.ID=GO:0090002; Evidence.code=IMP; Information.content=4.08 |
| ENSG00000132819 | SOURCE=GO.BP; GO.term.name=negative regulation of cell proliferation; GO.term.ID=GO:0008285; Evidence.code=IDA; Information.content=3.53 |
| ENSG00000132938 | SOURCE=PPI.MENTHA; SCORE=0.523; PMID=25416956 |
| ENSG00000133321 | SOURCE=GO.BP; GO.term.name=negative regulation of cell proliferation; GO.term.ID=GO:0008285; Evidence.code=TAS; Information.content=3.53 |
| ENSG00000133639 | SOURCE=GO.BP; GO.term.name=negative regulation of cell proliferation; GO.term.ID=GO:0008285; Evidence.code=IDA; Information.content=3.53 |
| ENSG00000133816 | SOURCE=GO.BP; GO.term.name=heart development; GO.term.ID=GO:0007507; Evidence.code=ISS; Information.content=3.25 |
| ENSG00000134250 | SOURCE=GO.BP; GO.term.name=negative regulation of cell proliferation; GO.term.ID=GO:0008285; Evidence.code=IDA; Information.content=3.53 |
| ENSG00000134308 | SOURCE=PPI.MENTHA; SCORE=0.21; PMID=12941695 |
| ENSG00000134318 | SOURCE=GO.BP; GO.term.name=establishment of protein localization to plasma membrane; GO.term.ID=GO:0090002; Evidence.code=IGI; Information.content=4.08; SOURCE=GO.BP; GO.term.name=establishment of protein localization to plasma membrane; GO.term.ID=GO:0090002; Evidence.code=IMP; Information.content=4.08 |
| ENSG00000134371 | SOURCE=GO.BP; GO.term.name=negative regulation of cell proliferation; GO.term.ID=GO:0008285; Evidence.code=IDA; Information.content=3.53 |
| ENSG00000134755 | SOURCE=PPI.STRING; SCORE=965; ORG_ENSP=ENSP00000280904; SOURCE=PPI.MENTHA; SCORE=0.309; PMID=11790773 ; SOURCE=GO.BP; GO.term.name=regulation of heart rate by cardiac conduction; GO.term.ID=GO:0086091; Evidence.code=IMP; Information.content=5 |
| ENSG00000134765 | SOURCE=PPI.STRING; SCORE=901; ORG_ENSP=ENSP00000257198; SOURCE=PPI.MENTHA; SCORE=0.309; PMID=11790773 |
| ENSG00000134817 | SOURCE=GO.BP; GO.term.name=heart development; GO.term.ID=GO:0007507; Evidence.code=ISS; Information.content=3.25 |
| ENSG00000134954 | SOURCE=GO.BP; GO.term.name=negative regulation of cell proliferation; GO.term.ID=GO:0008285; Evidence.code=TAS; Information.content=3.53 |
| ENSG00000135269 | SOURCE=GO.BP; GO.term.name=negative regulation of cell proliferation; GO.term.ID=GO:0008285; Evidence.code=IMP; Information.content=3.53 |
| ENSG00000135535 | SOURCE=GO.BP; GO.term.name=negative regulation of cell proliferation; GO.term.ID=GO:0008285; Evidence.code=NAS; Information.content=3.53; SOURCE=GO.BP; GO.term.name=negative regulation of cell proliferation; GO.term.ID=GO:0008285; Evidence.code=TAS; Information.content=3.53 |
| ENSG00000135956 | SOURCE=GO.BP; GO.term.name=negative regulation of cell proliferation; GO.term.ID=GO:0008285; Evidence.code=IMP; Information.content=3.53 |
| ENSG00000136235 | SOURCE=GO.BP; GO.term.name=negative regulation of cell proliferation; GO.term.ID=GO:0008285; Evidence.code=TAS; Information.content=3.53 |
| ENSG00000136383 | SOURCE=GO.BP; GO.term.name=heart development; GO.term.ID=GO:0007507; Evidence.code=ISS; Information.content=3.25 |
| ENSG00000136754 | SOURCE=GO.BP; GO.term.name=negative regulation of cell proliferation; GO.term.ID=GO:0008285; Evidence.code=TAS; Information.content=3.53 |
| ENSG00000136826 | SOURCE=GO.BP; GO.term.name=negative regulation of cell proliferation; GO.term.ID=GO:0008285; Evidence.code=TAS; Information.content=3.53 |
| ENSG00000136848 | SOURCE=GO.BP; GO.term.name=negative regulation of cell proliferation; GO.term.ID=GO:0008285; Evidence.code=IDA; Information.content=3.53 |
| ENSG00000137309 | SOURCE=GO.BP; GO.term.name=negative regulation of cell proliferation; GO.term.ID=GO:0008285; Evidence.code=IMP; Information.content=3.53 |
| ENSG00000137312 | SOURCE=GO.BP; GO.term.name=establishment of protein localization to plasma membrane; GO.term.ID=GO:0090002; Evidence.code=IMP; Information.content=4.08 |
| ENSG00000137492 | SOURCE=GO.BP; GO.term.name=negative regulation of cell proliferation; GO.term.ID=GO:0008285; Evidence.code=TAS; Information.content=3.53 |
| ENSG00000137573 | SOURCE=GO.BP; GO.term.name=negative regulation of cell migration; GO.term.ID=GO:0030336; Evidence.code=IMP; Information.content=4.04 |
| ENSG00000137710 | SOURCE=GO.BP; GO.term.name=establishment of protein localization to plasma membrane; GO.term.ID=GO:0090002; Evidence.code=IMP; Information.content=4.08 |
| ENSG00000137947 | SOURCE=PPI.MENTHA; SCORE=0.21; PMID=11416169 |
| ENSG00000138061 | SOURCE=GO.BP; GO.term.name=negative regulation of cell proliferation; GO.term.ID=GO:0008285; Evidence.code=ISS; Information.content=3.53; SOURCE=GO.BP; GO.term.name=negative regulation of cell migration; GO.term.ID=GO:0030336; Evidence.code=ISS; Information.content=4.04 |
| ENSG00000138193 | SOURCE=GO.BP; GO.term.name=heart development; GO.term.ID=GO:0007507; Evidence.code=TAS; Information.content=3.25 |
| ENSG00000138207 | SOURCE=GO.BP; GO.term.name=heart development; GO.term.ID=GO:0007507; Evidence.code=ISS; Information.content=3.25 |
| ENSG00000139567 | SOURCE=GO.BP; GO.term.name=negative regulation of cell proliferation; GO.term.ID=GO:0008285; Evidence.code=IMP; Information.content=3.53; SOURCE=GO.BP; GO.term.name=negative regulation of cell migration; GO.term.ID=GO:0030336; Evidence.code=IMP; Information.content=4.04 |
| ENSG00000139679 | SOURCE=COMPLEX.BIOPLEX; SCORE=0.99084065; ORG_UNIPROT=10161; SOURCE=PPI.MENTHA; SCORE=0.126; PMID=26186194 |
| ENSG00000139842 | SOURCE=GO.BP; GO.term.name=negative regulation of cell proliferation; GO.term.ID=GO:0008285; Evidence.code=TAS; Information.content=3.53 |
| ENSG00000140105 | SOURCE=GO.BP; GO.term.name=negative regulation of cell proliferation; GO.term.ID=GO:0008285; Evidence.code=TAS; Information.content=3.53 |
| ENSG00000140374 | SOURCE=GO.BP; GO.term.name=lipid homeostasis; GO.term.ID=GO:0055088; Evidence.code=IBA; Information.content=3.95 |
| ENSG00000140416 | SOURCE=GO.BP; GO.term.name=negative regulation of cell migration; GO.term.ID=GO:0030336; Evidence.code=ISS; Information.content=4.04 |
| ENSG00000140464 | SOURCE=GO.BP; GO.term.name=negative regulation of cell proliferation; GO.term.ID=GO:0008285; Evidence.code=IMP; Information.content=3.53 |
| ENSG00000140682 | SOURCE=GO.BP; GO.term.name=negative regulation of cell proliferation; GO.term.ID=GO:0008285; Evidence.code=TAS; Information.content=3.53 |
| ENSG00000140945 | SOURCE=GO.BP; GO.term.name=negative regulation of cell proliferation; GO.term.ID=GO:0008285; Evidence.code=IDA; Information.content=3.53 |
| ENSG00000141002 | SOURCE=GO.BP; GO.term.name=heart development; GO.term.ID=GO:0007507; Evidence.code=NAS; Information.content=3.25 |
| ENSG00000141013 | SOURCE=GO.BP; GO.term.name=negative regulation of cell proliferation; GO.term.ID=GO:0008285; Evidence.code=TAS; Information.content=3.53 |
| ENSG00000141052 | SOURCE=GO.BP; GO.term.name=negative regulation of cell proliferation; GO.term.ID=GO:0008285; Evidence.code=IDA; Information.content=3.53 |
| ENSG00000141232 | SOURCE=GO.BP; GO.term.name=negative regulation of cell proliferation; GO.term.ID=GO:0008285; Evidence.code=IDA; Information.content=3.53; SOURCE=GO.BP; GO.term.name=negative regulation of cell proliferation; GO.term.ID=GO:0008285; Evidence.code=TAS; Information.content=3.53 |
| ENSG00000141736 | SOURCE=PPI.MENTHA; SCORE=0.126; PMID=24189400 |
| ENSG00000142227 | SOURCE=GO.BP; GO.term.name=negative regulation of cell proliferation; GO.term.ID=GO:0008285; Evidence.code=TAS; Information.content=3.53 |
| ENSG00000143153 | SOURCE=GO.BP; GO.term.name=cell communication by electrical coupling involved in cardiac conduction; GO.term.ID=GO:0086064; Evidence.code=TAS; Information.content=5.53 |
| ENSG00000143499 | SOURCE=GO.BP; GO.term.name=negative regulation of cell proliferation; GO.term.ID=GO:0008285; Evidence.code=ISS; Information.content=3.53 |
| ENSG00000143537 | SOURCE=GO.BP; GO.term.name=negative regulation of cell migration; GO.term.ID=GO:0030336; Evidence.code=IMP; Information.content=4.04 |
| ENSG00000143545 | SOURCE=GO.BP; GO.term.name=establishment of protein localization to plasma membrane; GO.term.ID=GO:0090002; Evidence.code=IMP; Information.content=4.08 |
| ENSG00000143816 | SOURCE=GO.BP; GO.term.name=negative regulation of cell proliferation; GO.term.ID=GO:0008285; Evidence.code=IMP; Information.content=3.53 |
| ENSG00000143867 | SOURCE=GO.BP; GO.term.name=heart development; GO.term.ID=GO:0007507; Evidence.code=ISS; Information.content=3.25 |
| ENSG00000143878 | SOURCE=GO.BP; GO.term.name=negative regulation of cell migration; GO.term.ID=GO:0030336; Evidence.code=IDA; Information.content=4.04 |
| ENSG00000144283 | SOURCE=Ens86Paralog; IDENTITY=18 |
| ENSG00000144535 | SOURCE=GO.BP; GO.term.name=negative regulation of cell proliferation; GO.term.ID=GO:0008285; Evidence.code=IMP; Information.content=3.53 |
| ENSG00000145349 | SOURCE=GO.BP; GO.term.name=regulation of heart rate by cardiac conduction; GO.term.ID=GO:0086091; Evidence.code=IC; Information.content=5 |
| ENSG00000145362 | SOURCE=GO.BP; GO.term.name=regulation of heart rate by cardiac conduction; GO.term.ID=GO:0086091; Evidence.code=IMP; Information.content=5; SOURCE=GO.BP; GO.term.name=regulation of heart rate by cardiac conduction; GO.term.ID=GO:0086091; Evidence.code=ISS; Information.content=5 |
| ENSG00000146674 | SOURCE=GO.BP; GO.term.name=negative regulation of cell proliferation; GO.term.ID=GO:0008285; Evidence.code=IGI; Information.content=3.53 |
| ENSG00000147162 | SOURCE=PPI.STRING; SCORE=736; ORG_ENSP=ENSP00000362824 |
| ENSG00000148396 | SOURCE=PPI.MENTHA; SCORE=0.236; PMID=26496610 |
| ENSG00000148411 | SOURCE=GO.BP; GO.term.name=negative regulation of cell proliferation; GO.term.ID=GO:0008285; Evidence.code=IDA; Information.content=3.53 |
| ENSG00000149575 | SOURCE=GO.BP; GO.term.name=regulation of heart rate by cardiac conduction; GO.term.ID=GO:0086091; Evidence.code=IMP; Information.content=5 |
| ENSG00000151067 | SOURCE=GO.BP; GO.term.name=heart development; GO.term.ID=GO:0007507; Evidence.code=IMP; Information.content=3.25; SOURCE=GO.BP; GO.term.name=cell communication by electrical coupling involved in cardiac conduction; GO.term.ID=GO:0086064; Evidence.code=TAS; Information.content=5.53; SOURCE=GO.BP; GO.term.name=regulation of heart rate by cardiac conduction; GO.term.ID=GO:0086091; Evidence.code=IMP; Information.content=5 |
| ENSG00000151150 | SOURCE=GO.BP; GO.term.name=positive regulation of sodium ion transport; GO.term.ID=GO:0010765; Evidence.code=ISS; Information.content=4.9 |
| ENSG00000151498 | SOURCE=GO.BP; GO.term.name=lipid homeostasis; GO.term.ID=GO:0055088; Evidence.code=IBA; Information.content=3.95 |
| ENSG00000152642 | SOURCE=GO.BP; GO.term.name=positive regulation of sodium ion transport; GO.term.ID=GO:0010765; Evidence.code=IMP; Information.content=4.9 |
| ENSG00000152661 | SOURCE=PPI.STRING; SCORE=925; ORG_ENSP=ENSP00000282561; SOURCE=GO.BP; GO.term.name=heart development; GO.term.ID=GO:0007507; Evidence.code=TAS; Information.content=3.25; SOURCE=GO.BP; GO.term.name=cell communication by electrical coupling involved in cardiac conduction; GO.term.ID=GO:0086064; Evidence.code=NAS; Information.content=5.53 |
| ENSG00000152894 | SOURCE=GO.BP; GO.term.name=negative regulation of cell proliferation; GO.term.ID=GO:0008285; Evidence.code=IDA; Information.content=3.53; SOURCE=GO.BP; GO.term.name=negative regulation of cell migration; GO.term.ID=GO:0030336; Evidence.code=IDA; Information.content=4.04 |
| ENSG00000153721 | SOURCE=GO.BP; GO.term.name=positive regulation of sodium ion transport; GO.term.ID=GO:0010765; Evidence.code=ISS; Information.content=4.9 |
| ENSG00000153956 | SOURCE=GO.BP; GO.term.name=regulation of heart rate by cardiac conduction; GO.term.ID=GO:0086091; Evidence.code=IMP; Information.content=5 |
| ENSG00000154096 | SOURCE=GO.BP; GO.term.name=negative regulation of cell migration; GO.term.ID=GO:0030336; Evidence.code=IBA; Information.content=4.04; SOURCE=GO.BP; GO.term.name=negative regulation of cell migration; GO.term.ID=GO:0030336; Evidence.code=ISS; Information.content=4.04 |
| ENSG00000154305 | SOURCE=GO.BP; GO.term.name=negative regulation of cell migration; GO.term.ID=GO:0030336; Evidence.code=IDA; Information.content=4.04 |
| ENSG00000154640 | SOURCE=GO.BP; GO.term.name=negative regulation of cell proliferation; GO.term.ID=GO:0008285; Evidence.code=TAS; Information.content=3.53 |
| ENSG00000154734 | SOURCE=GO.BP; GO.term.name=negative regulation of cell proliferation; GO.term.ID=GO:0008285; Evidence.code=TAS; Information.content=3.53 |
| ENSG00000154803 | SOURCE=GO.BP; GO.term.name=negative regulation of cell migration; GO.term.ID=GO:0030336; Evidence.code=IMP; Information.content=4.04 |
| ENSG00000155090 | SOURCE=GO.BP; GO.term.name=negative regulation of cell proliferation; GO.term.ID=GO:0008285; Evidence.code=TAS; Information.content=3.53 |
| ENSG00000155508 | SOURCE=GO.BP; GO.term.name=negative regulation of cell proliferation; GO.term.ID=GO:0008285; Evidence.code=TAS; Information.content=3.53 |
| ENSG00000155827 | SOURCE=GO.BP; GO.term.name=negative regulation of cell migration; GO.term.ID=GO:0030336; Evidence.code=IDA; Information.content=4.04 |
| ENSG00000156795 | SOURCE=COMPLEX.BIOPLEX; SCORE=0.958358177; ORG_UNIPROT=55093 |
| ENSG00000157933 | SOURCE=GO.BP; GO.term.name=negative regulation of cell proliferation; GO.term.ID=GO:0008285; Evidence.code=IDA; Information.content=3.53 |
| ENSG00000158286 | SOURCE=GO.BP; GO.term.name=cell-cell signaling involved in cardiac conduction; GO.term.ID=GO:0086019; Evidence.code=ISS; Information.content=4.9 |
| ENSG00000158773 | SOURCE=GO.BP; GO.term.name=lipid homeostasis; GO.term.ID=GO:0055088; Evidence.code=ISS; Information.content=3.95 |
| ENSG00000159388 | SOURCE=GO.BP; GO.term.name=negative regulation of cell proliferation; GO.term.ID=GO:0008285; Evidence.code=IMP; Information.content=3.53; SOURCE=GO.BP; GO.term.name=negative regulation of cell proliferation; GO.term.ID=GO:0008285; Evidence.code=TAS; Information.content=3.53 |
| ENSG00000159692 | SOURCE=GO.BP; GO.term.name=negative regulation of cell proliferation; GO.term.ID=GO:0008285; Evidence.code=TAS; Information.content=3.53 |
| ENSG00000160007 | SOURCE=PPI.STRING; SCORE=719; ORG_ENSP=ENSP00000385720 |
| ENSG00000160862 | SOURCE=GO.BP; GO.term.name=negative regulation of cell proliferation; GO.term.ID=GO:0008285; Evidence.code=NAS; Information.content=3.53 |
| ENSG00000161533 | SOURCE=GO.BP; GO.term.name=lipid homeostasis; GO.term.ID=GO:0055088; Evidence.code=IDA; Information.content=3.95; SOURCE=GO.BP; GO.term.name=lipid homeostasis; GO.term.ID=GO:0055088; Evidence.code=IGI; Information.content=3.95 |
| ENSG00000162521 | SOURCE=GO.BP; GO.term.name=negative regulation of cell proliferation; GO.term.ID=GO:0008285; Evidence.code=TAS; Information.content=3.53 |
| ENSG00000162998 | SOURCE=GO.BP; GO.term.name=negative regulation of cell proliferation; GO.term.ID=GO:0008285; Evidence.code=IDA; Information.content=3.53 |
| ENSG00000163191 | SOURCE=GO.BP; GO.term.name=negative regulation of cell proliferation; GO.term.ID=GO:0008285; Evidence.code=TAS; Information.content=3.53 |
| ENSG00000163217 | SOURCE=GO.BP; GO.term.name=negative regulation of cell migration; GO.term.ID=GO:0030336; Evidence.code=IDA; Information.content=4.04 |
| ENSG00000163399 | SOURCE=GO.BP; GO.term.name=cell communication by electrical coupling involved in cardiac conduction; GO.term.ID=GO:0086064; Evidence.code=TAS; Information.content=5.53 |
| ENSG00000163453 | SOURCE=GO.BP; GO.term.name=negative regulation of cell proliferation; GO.term.ID=GO:0008285; Evidence.code=TAS; Information.content=3.53 |
| ENSG00000163513 | SOURCE=GO.BP; GO.term.name=heart development; GO.term.ID=GO:0007507; Evidence.code=ISS; Information.content=3.25 |
| ENSG00000163539 | SOURCE=GO.BP; GO.term.name=establishment of protein localization to plasma membrane; GO.term.ID=GO:0090002; Evidence.code=IMP; Information.content=4.08 |
| ENSG00000163930 | SOURCE=GO.BP; GO.term.name=negative regulation of cell proliferation; GO.term.ID=GO:0008285; Evidence.code=TAS; Information.content=3.53 |
| ENSG00000163939 | SOURCE=GO.BP; GO.term.name=negative regulation of cell proliferation; GO.term.ID=GO:0008285; Evidence.code=IMP; Information.content=3.53 |
| ENSG00000164107 | SOURCE=GO.BP; GO.term.name=heart development; GO.term.ID=GO:0007507; Evidence.code=NAS; Information.content=3.25 |
| ENSG00000164442 | SOURCE=GO.BP; GO.term.name=heart development; GO.term.ID=GO:0007507; Evidence.code=IMP; Information.content=3.25; SOURCE=GO.BP; GO.term.name=heart development; GO.term.ID=GO:0007507; Evidence.code=ISS; Information.content=3.25; SOURCE=GO.BP; GO.term.name=negative regulation of cell migration; GO.term.ID=GO:0030336; Evidence.code=IMP; Information.content=4.04 |
| ENSG00000164741 | SOURCE=GO.BP; GO.term.name=negative regulation of cell proliferation; GO.term.ID=GO:0008285; Evidence.code=IDA; Information.content=3.53; SOURCE=GO.BP; GO.term.name=negative regulation of cell proliferation; GO.term.ID=GO:0008285; Evidence.code=IMP; Information.content=3.53; SOURCE=GO.BP; GO.term.name=negative regulation of cell migration; GO.term.ID=GO:0030336; Evidence.code=IDA; Information.content=4.04; SOURCE=GO.BP; GO.term.name=negative regulation of cell migration; GO.term.ID=GO:0030336; Evidence.code=IMP; Information.content=4.04 |
| ENSG00000164924 | SOURCE=PPI.MENTHA; SCORE=0.21; PMID=12941695 |
| ENSG00000164938 | SOURCE=GO.BP; GO.term.name=negative regulation of cell proliferation; GO.term.ID=GO:0008285; Evidence.code=ISS; Information.content=3.53; SOURCE=GO.BP; GO.term.name=negative regulation of cell migration; GO.term.ID=GO:0030336; Evidence.code=ISS; Information.content=4.04 |
| ENSG00000165238 | SOURCE=GO.BP; GO.term.name=negative regulation of cell proliferation; GO.term.ID=GO:0008285; Evidence.code=IMP; Information.content=3.53 |
| ENSG00000165699 | SOURCE=GO.BP; GO.term.name=negative regulation of cell proliferation; GO.term.ID=GO:0008285; Evidence.code=IMP; Information.content=3.53 |
| ENSG00000165914 | SOURCE=GO.BP; GO.term.name=establishment of protein localization to plasma membrane; GO.term.ID=GO:0090002; Evidence.code=IDA; Information.content=4.08 |
| ENSG00000165995 | SOURCE=GO.BP; GO.term.name=regulation of heart rate by cardiac conduction; GO.term.ID=GO:0086091; Evidence.code=IMP; Information.content=5; SOURCE=GO.BP; GO.term.name=establishment of protein localization to plasma membrane; GO.term.ID=GO:0090002; Evidence.code=ISS; Information.content=4.08 |
| ENSG00000166147 | SOURCE=GO.BP; GO.term.name=heart development; GO.term.ID=GO:0007507; Evidence.code=IMP; Information.content=3.25 |
| ENSG00000166266 | SOURCE=GO.BP; GO.term.name=negative regulation of cell proliferation; GO.term.ID=GO:0008285; Evidence.code=TAS; Information.content=3.53 |
| ENSG00000166333 | SOURCE=PPI.MENTHA; SCORE=0.126; PMID=25852190 |
| ENSG00000166579 | SOURCE=PPI.STRING; SCORE=771; ORG_ENSP=ENSP00000333982; SOURCE=PPI.MENTHA; SCORE=0.523; PMID=25416956 |
| ENSG00000166913 | SOURCE=PPI.MENTHA; SCORE=0.236; PMID=24255178 |
| ENSG00000167085 | SOURCE=GO.BP; GO.term.name=negative regulation of cell proliferation; GO.term.ID=GO:0008285; Evidence.code=IMP; Information.content=3.53 |
| ENSG00000167996 | SOURCE=GO.BP; GO.term.name=negative regulation of cell proliferation; GO.term.ID=GO:0008285; Evidence.code=ISS; Information.content=3.53 |
| ENSG00000168036 | SOURCE=PPI.STRING; SCORE=805; ORG_ENSP=ENSP00000344456; SOURCE=GO.BP; GO.term.name=negative regulation of cell proliferation; GO.term.ID=GO:0008285; Evidence.code=IDA; Information.content=3.53 |
| ENSG00000168283 | SOURCE=PPI.MENTHA; SCORE=0.126; PMID=24457600 |
| ENSG00000168306 | SOURCE=GO.BP; GO.term.name=lipid homeostasis; GO.term.ID=GO:0055088; Evidence.code=IBA; Information.content=3.95 |
| ENSG00000168542 | SOURCE=GO.BP; GO.term.name=heart development; GO.term.ID=GO:0007507; Evidence.code=IMP; Information.content=3.25 |
| ENSG00000168675 | SOURCE=GO.BP; GO.term.name=negative regulation of cell migration; GO.term.ID=GO:0030336; Evidence.code=IMP; Information.content=4.04 |
| ENSG00000168710 | SOURCE=GO.BP; GO.term.name=positive regulation of sodium ion transport; GO.term.ID=GO:0010765; Evidence.code=IMP; Information.content=4.9 |
| ENSG00000168890 | SOURCE=GO.BP; GO.term.name=establishment of protein localization to plasma membrane; GO.term.ID=GO:0090002; Evidence.code=IDA; Information.content=4.08 |
| ENSG00000169032 | SOURCE=GO.BP; GO.term.name=negative regulation of cell proliferation; GO.term.ID=GO:0008285; Evidence.code=IDA; Information.content=3.53 |
| ENSG00000169083 | SOURCE=GO.BP; GO.term.name=negative regulation of cell proliferation; GO.term.ID=GO:0008285; Evidence.code=IMP; Information.content=3.53 |
| ENSG00000169504 | SOURCE=GO.BP; GO.term.name=negative regulation of cell migration; GO.term.ID=GO:0030336; Evidence.code=IDA; Information.content=4.04 |
| ENSG00000170345 | SOURCE=PPI.MENTHA; SCORE=0.126; PMID=20195357 |
| ENSG00000170776 | SOURCE=GO.BP; GO.term.name=heart development; GO.term.ID=GO:0007507; Evidence.code=ISS; Information.content=3.25 |
| ENSG00000170876 | SOURCE=PPI.STRING; SCORE=730; ORG_ENSP=ENSP00000303992 |
| ENSG00000170881 | SOURCE=GO.BP; GO.term.name=negative regulation of cell proliferation; GO.term.ID=GO:0008285; Evidence.code=IDA; Information.content=3.53 |
| ENSG00000171056 | SOURCE=GO.BP; GO.term.name=negative regulation of cell proliferation; GO.term.ID=GO:0008285; Evidence.code=IDA; Information.content=3.53 |
| ENSG00000171862 | SOURCE=GO.BP; GO.term.name=heart development; GO.term.ID=GO:0007507; Evidence.code=ISS; Information.content=3.25; SOURCE=GO.BP; GO.term.name=negative regulation of cell proliferation; GO.term.ID=GO:0008285; Evidence.code=IDA; Information.content=3.53; SOURCE=GO.BP; GO.term.name=negative regulation of cell proliferation; GO.term.ID=GO:0008285; Evidence.code=IMP; Information.content=3.53; SOURCE=GO.BP; GO.term.name=negative regulation of cell migration; GO.term.ID=GO:0030336; Evidence.code=IMP; Information.content=4.04 |
| ENSG00000172059 | SOURCE=GO.BP; GO.term.name=negative regulation of cell proliferation; GO.term.ID=GO:0008285; Evidence.code=IDA; Information.content=3.53; SOURCE=GO.BP; GO.term.name=negative regulation of cell proliferation; GO.term.ID=GO:0008285; Evidence.code=TAS; Information.content=3.53 |
| ENSG00000172819 | SOURCE=GO.BP; GO.term.name=negative regulation of cell proliferation; GO.term.ID=GO:0008285; Evidence.code=ISS; Information.content=3.53 |
| ENSG00000173402 | SOURCE=GO.BP; GO.term.name=negative regulation of cell migration; GO.term.ID=GO:0030336; Evidence.code=IMP; Information.content=4.04 |
| ENSG00000173801 | SOURCE=PPI.STRING; SCORE=921; ORG_ENSP=ENSP00000311113; SOURCE=PPI.MENTHA; SCORE=0.454; PMID=11790773 ; SOURCE=GO.BP; GO.term.name=desmosome assembly; GO.term.ID=GO:0002159; Evidence.code=IDA; Information.content=5.44; SOURCE=GO.BP; GO.term.name=desmosome assembly; GO.term.ID=GO:0002159; Evidence.code=IMP; Information.content=5.44; SOURCE=GO.BP; GO.term.name=regulation of heart rate by cardiac conduction; GO.term.ID=GO:0086091; Evidence.code=IMP; Information.content=5; SOURCE=GO.BP; GO.term.name=establishment of protein localization to plasma membrane; GO.term.ID=GO:0090002; Evidence.code=IMP; Information.content=4.08 |
| ENSG00000174173 | SOURCE=PPI.MENTHA; SCORE=0.21; PMID=18984158 |
| ENSG00000174348 | SOURCE=GO.BP; GO.term.name=negative regulation of cell proliferation; GO.term.ID=GO:0008285; Evidence.code=IDA; Information.content=3.53; SOURCE=GO.BP; GO.term.name=negative regulation of cell migration; GO.term.ID=GO:0030336; Evidence.code=IDA; Information.content=4.04 |
| ENSG00000174738 | SOURCE=GO.BP; GO.term.name=lipid homeostasis; GO.term.ID=GO:0055088; Evidence.code=ISS; Information.content=3.95 |
| ENSG00000174775 | SOURCE=GO.BP; GO.term.name=negative regulation of cell proliferation; GO.term.ID=GO:0008285; Evidence.code=IDA; Information.content=3.53 |
| ENSG00000175029 | SOURCE=GO.BP; GO.term.name=negative regulation of cell proliferation; GO.term.ID=GO:0008285; Evidence.code=TAS; Information.content=3.53 |
| ENSG00000175274 | SOURCE=GO.BP; GO.term.name=negative regulation of cell proliferation; GO.term.ID=GO:0008285; Evidence.code=TAS; Information.content=3.53 |
| ENSG00000177098 | SOURCE=GO.BP; GO.term.name=positive regulation of sodium ion transport; GO.term.ID=GO:0010765; Evidence.code=IDA; Information.content=4.9; SOURCE=GO.BP; GO.term.name=regulation of heart rate by cardiac conduction; GO.term.ID=GO:0086091; Evidence.code=IMP; Information.content=5 |
| ENSG00000177646 | SOURCE=GO.BP; GO.term.name=lipid homeostasis; GO.term.ID=GO:0055088; Evidence.code=IBA; Information.content=3.95 |
| ENSG00000178252 | SOURCE=GO.BP; GO.term.name=negative regulation of cell proliferation; GO.term.ID=GO:0008285; Evidence.code=IDA; Information.content=3.53 |
| ENSG00000179295 | SOURCE=GO.BP; GO.term.name=heart development; GO.term.ID=GO:0007507; Evidence.code=IMP; Information.content=3.25 |
| ENSG00000180228 | SOURCE=GO.BP; GO.term.name=negative regulation of cell proliferation; GO.term.ID=GO:0008285; Evidence.code=TAS; Information.content=3.53 |
| ENSG00000180573 | SOURCE=GO.BP; GO.term.name=negative regulation of cell proliferation; GO.term.ID=GO:0008285; Evidence.code=IMP; Information.content=3.53 |
| ENSG00000181104 | SOURCE=GO.BP; GO.term.name=negative regulation of cell proliferation; GO.term.ID=GO:0008285; Evidence.code=IDA; Information.content=3.53 |
| ENSG00000181163 | SOURCE=GO.BP; GO.term.name=negative regulation of cell proliferation; GO.term.ID=GO:0008285; Evidence.code=IMP; Information.content=3.53; SOURCE=GO.BP; GO.term.name=negative regulation of cell proliferation; GO.term.ID=GO:0008285; Evidence.code=ISS; Information.content=3.53 |
| ENSG00000181852 | SOURCE=GO.BP; GO.term.name=negative regulation of cell proliferation; GO.term.ID=GO:0008285; Evidence.code=IDA; Information.content=3.53; SOURCE=GO.BP; GO.term.name=negative regulation of cell migration; GO.term.ID=GO:0030336; Evidence.code=IMP; Information.content=4.04 |
| ENSG00000182636 | SOURCE=GO.BP; GO.term.name=negative regulation of cell proliferation; GO.term.ID=GO:0008285; Evidence.code=TAS; Information.content=3.53 |
| ENSG00000182871 | SOURCE=GO.BP; GO.term.name=negative regulation of cell proliferation; GO.term.ID=GO:0008285; Evidence.code=TAS; Information.content=3.53 |
| ENSG00000183023 | SOURCE=GO.BP; GO.term.name=cell communication by electrical coupling involved in cardiac conduction; GO.term.ID=GO:0086064; Evidence.code=ISS; Information.content=5.53 |
| ENSG00000183072 | SOURCE=GO.BP; GO.term.name=positive regulation of sodium ion transport; GO.term.ID=GO:0010765; Evidence.code=ISS; Information.content=4.9 |
| ENSG00000183230 | SOURCE=GO.BP; GO.term.name=regulation of heart rate by cardiac conduction; GO.term.ID=GO:0086091; Evidence.code=IMP; Information.content=5 |
| ENSG00000183337 | SOURCE=GO.BP; GO.term.name=heart development; GO.term.ID=GO:0007507; Evidence.code=IMP; Information.content=3.25 |
| ENSG00000183864 | SOURCE=GO.BP; GO.term.name=negative regulation of cell proliferation; GO.term.ID=GO:0008285; Evidence.code=TAS; Information.content=3.53 |
| ENSG00000183873 | SOURCE=PPI.STRING; SCORE=903; ORG_ENSP=ENSP00000328968; SOURCE=GO.BP; GO.term.name=positive regulation of sodium ion transport; GO.term.ID=GO:0010765; Evidence.code=IDA; Information.content=4.9; SOURCE=GO.BP; GO.term.name=regulation of heart rate by cardiac conduction; GO.term.ID=GO:0086091; Evidence.code=IMP; Information.content=5 |
| ENSG00000184481 | SOURCE=GO.BP; GO.term.name=negative regulation of cell proliferation; GO.term.ID=GO:0008285; Evidence.code=IDA; Information.content=3.53 |
| ENSG00000185043 | SOURCE=GO.BP; GO.term.name=negative regulation of cell proliferation; GO.term.ID=GO:0008285; Evidence.code=IMP; Information.content=3.53 |
| ENSG00000185359 | SOURCE=GO.BP; GO.term.name=negative regulation of cell proliferation; GO.term.ID=GO:0008285; Evidence.code=TAS; Information.content=3.53 |
| ENSG00000185885 | SOURCE=GO.BP; GO.term.name=negative regulation of cell proliferation; GO.term.ID=GO:0008285; Evidence.code=IMP; Information.content=3.53; SOURCE=GO.BP; GO.term.name=negative regulation of cell proliferation; GO.term.ID=GO:0008285; Evidence.code=TAS; Information.content=3.53; SOURCE=GO.BP; GO.term.name=negative regulation of cell migration; GO.term.ID=GO:0030336; Evidence.code=IMP; Information.content=4.04 |
| ENSG00000186575 | SOURCE=GO.BP; GO.term.name=negative regulation of cell proliferation; GO.term.ID=GO:0008285; Evidence.code=IDA; Information.content=3.53; SOURCE=GO.BP; GO.term.name=negative regulation of cell proliferation; GO.term.ID=GO:0008285; Evidence.code=IMP; Information.content=3.53; SOURCE=GO.BP; GO.term.name=negative regulation of cell migration; GO.term.ID=GO:0030336; Evidence.code=TAS; Information.content=4.04 |
| ENSG00000196177 | SOURCE=GO.BP; GO.term.name=lipid homeostasis; GO.term.ID=GO:0055088; Evidence.code=IBA; Information.content=3.95 |
| ENSG00000196712 | SOURCE=GO.BP; GO.term.name=heart development; GO.term.ID=GO:0007507; Evidence.code=ISS; Information.content=3.25; SOURCE=GO.BP; GO.term.name=negative regulation of cell migration; GO.term.ID=GO:0030336; Evidence.code=IMP; Information.content=4.04 |
| ENSG00000197256 | SOURCE=GO.BP; GO.term.name=negative regulation of cell proliferation; GO.term.ID=GO:0008285; Evidence.code=IDA; Information.content=3.53 |
| ENSG00000197747 | SOURCE=GO.BP; GO.term.name=establishment of protein localization to plasma membrane; GO.term.ID=GO:0090002; Evidence.code=IDA; Information.content=4.08 |
| ENSG00000197879 | SOURCE=GO.BP; GO.term.name=regulation of bicellular tight junction assembly; GO.term.ID=GO:2000810; Evidence.code=IMP; Information.content=5.33 |
| ENSG00000198420 | SOURCE=GO.BP; GO.term.name=negative regulation of cell migration; GO.term.ID=GO:0030336; Evidence.code=IDA; Information.content=4.04 |
| ENSG00000198561 | SOURCE=Ens86Paralog; IDENTITY=20 |
| ENSG00000198612 | SOURCE=GO.BP; GO.term.name=negative regulation of cell proliferation; GO.term.ID=GO:0008285; Evidence.code=IMP; Information.content=3.53 |
| ENSG00000198626 | SOURCE=GO.BP; GO.term.name=cell communication by electrical coupling involved in cardiac conduction; GO.term.ID=GO:0086064; Evidence.code=IC; Information.content=5.53 |
| ENSG00000198791 | SOURCE=GO.BP; GO.term.name=negative regulation of cell proliferation; GO.term.ID=GO:0008285; Evidence.code=IDA; Information.content=3.53 |
| ENSG00000198947 | SOURCE=GO.BP; GO.term.name=cardiac muscle cell action potential; GO.term.ID=GO:0086001; Evidence.code=ISS; Information.content=4.5 |
| ENSG00000203867 | SOURCE=GO.BP; GO.term.name=heart development; GO.term.ID=GO:0007507; Evidence.code=IMP; Information.content=3.25 |
| ENSG00000204435 | SOURCE=GO.BP; GO.term.name=negative regulation of cell proliferation; GO.term.ID=GO:0008285; Evidence.code=TAS; Information.content=3.53 |
| ENSG00000205336 | SOURCE=GO.BP; GO.term.name=negative regulation of cell proliferation; GO.term.ID=GO:0008285; Evidence.code=IDA; Information.content=3.53 |
| ENSG00000213024 | SOURCE=GO.BP; GO.term.name=negative regulation of cell proliferation; GO.term.ID=GO:0008285; Evidence.code=IDA; Information.content=3.53; SOURCE=GO.BP; GO.term.name=negative regulation of cell proliferation; GO.term.ID=GO:0008285; Evidence.code=ISS; Information.content=3.53 |
| ENSG00000213145 | SOURCE=GO.BP; GO.term.name=heart development; GO.term.ID=GO:0007507; Evidence.code=TAS; Information.content=3.25 |
| ENSG00000213949 | SOURCE=GO.BP; GO.term.name=negative regulation of cell proliferation; GO.term.ID=GO:0008285; Evidence.code=IMP; Information.content=3.53 |
| ENSG00000239672 | SOURCE=GO.BP; GO.term.name=negative regulation of cell proliferation; GO.term.ID=GO:0008285; Evidence.code=TAS; Information.content=3.53 |
| ENSG00000254087 | SOURCE=GO.BP; GO.term.name=negative regulation of cell proliferation; GO.term.ID=GO:0008285; Evidence.code=IMP; Information.content=3.53 |
| ENSG00000254901 | SOURCE=GO.BP; GO.term.name=heart development; GO.term.ID=GO:0007507; Evidence.code=IEP; Information.content=3.25 |
| ENSG00000263155 | SOURCE=PPI.STRING; SCORE=833; ORG_ENSP=ENSP00000267853 |

SOURCE: Source of this gene for the PKP2-related get set. Either one of GO.BP (Gene Ontology Biological process annotation), PPI.STRING (protein protein interaction from the STRING database), PPI.MENTHA (protein protein interaction from the mentha database), Oreganno (transcription factor from the Orgeganno database), TR (transcription factor from the Ensembl database), COMPLEX.BIOPLEX (protein complex data from the Bioplex database), Ens86Paralog (paralog information from Ensembl)

For each source, additional information are given to the extend as they were available.

**Supplementary Table S3.**

| **Family** | **Gene set** | **Gene rank** | **dbSNP ID** | **Chr** | **Pos** | **Ref** | **Alt** | **Consequence** | **Gene** | **HGVSp** | **HGVSc** | **PROVEAN score** | **PROVEAN binary prediction** | **ExAC_AF** | **LR.PFS3 value** | **LR.PFS3 binary prediction** | **Gene expression (FPKM)** | **linkage** | **genotype** | **comment** | **PKP2 mechanism** | **PKP2 mechanism info** |
| --- | --- | --- | --- | --- | --- | --- | --- | --- | --- | --- | --- | --- | --- | --- | --- | --- | --- | --- | --- | --- | --- | --- |
| Fam2 | ACM | - | rs55886356 | 2 | 179399576 | C | G | missense | TTN | ENSP00000434586.1:p.Arg23483His | ENST00000460472.2:c.74571G>C | -4.32 | deleterious | 6.8x10-3 | 0.807 | pathogenic | 35.2 | - | het in Fam2.II.1 and Fam2.I.1 | Mutations in TTN can cause ACM [19]. | - | - |
| Fam2 | ACM | - | rs55676195 | 2 | 179406161 | C | T | missense | TTN | ENSP00000434586.1:p.Arg23483His | ENST00000460472.2:c.70448G>A | -3.67 | deleterious | 6.6x10-5 | 0.869 | pathogenic | 35.2 | - | het in Fam2.II.1 and Fam2.I.1 | Mutations in TTN can cause ACM [19]. | - | - |
| Fam2 | ACM | - | rs34070843 | 2 | 179605725 | T | C | missense | TTN | ENSP00000434586.1:p.Ile3716Val | ENST00000460472.2:c.11146A>G | -0.22 | neutral | 0.018 | 0.377 | benign | 35.2 | - | het in Fam2.II.1 and Fam2.I.1 | Mutations in TTN can cause ACM [19]. | - | - |
| Fam1 | PKP2 | 1 | rs145403829 | 3 | 49548225 | G | C | missense | DAG1 | ENSP00000312435.2:p.Leu86Phe | ENST00000308775.2:c.258G>C | -0.56 | neutral | 6.6x10-5 | 0.608 | pathogenic | 36.4 | - | het in Fam1.III.2, Fam1.III.3, Fam1.II.1 | β-dystroglycan binds to Hippo pathway effector Yap to inhibit cardiomyocyte proliferation in mice [20] | GO BP | negative regulation of cell migration (GO:0030336) |
| Fam1 | PKP2 | 2 | rs146419192 | 16 | 89975448 | C | T | missense | TCF25 | ENSP00000263347.7:p.Ser390Phe | ENST00000263347.7:c.1169C>T | -0.65 | neutral | 0.5x10-5 | NA | NA | 32.8 | - | het in Fam1.III.2, Fam1.III.3, Fam1.II.1 | Negatively regulates SRF, whose increased expression causes cardiomyopathy in mice [21] | GO BP | heart development (GO:0007507) |
| Fam1 | PKP2 | 3 | NA | 9 | 124329496 | A | G | missense | DAB2IP | ENSP00000259371.2:p.Asp10Gly | ENST00000259371.2:c.29A>G | -0.17 | neutral | NA | NA | NA | 12.6 | - | het in Fam1.III.2, Fam1.III.3, Fam1.II.1 | One variant in DAB2IP has been associated with coronary heart disease [22]. | GO BP | negative regulation of cell proliferation (GO:0008285) |
| Fam1 | PKP2 | 4 | rs41303611 | 10 | 126694170 | C | T | missense | CTBP2 | ENSP00000357816.5:p.Gly70Arg | ENST00000334808.6:c.208G>A | -0.623 | neutral | 0.016 | NA | NA | 6 | - | het in Fam1.III.2, Fam1.III.3, Fam1.II.1 | Ctbp2-null mice have defective heart morphogenesis. CTBP2 may be a regulator of Wnt-mediated gene expression [23]. | GO BP | negative regulation of cell proliferation (GO:0008285) |
| Fam2 | PKP2 | 1 | rs200736826 | 5 | 131820131 | T | C | missense | IRF1 | ENSP00000384406.1:p.Asn259Ser | ENST00000405885.2:c.776A>G | -2.89 | deleterious | 8.2x10-5 | 0.870 | pathogenic | 7.2 | - | het in Fam2.II.1 and Fam2.I.1 | IRF1 is associated with cancer and a negative regulator of coronary artery smooth muscle cells (OMIM *147575) [16] | GO BP | negative regulation of cell proliferation (GO:0008285) |
| Fam2 | PKP2 | 2 | rs140604493 | 17 | 1373742 | G | T | missense | MYO1C | ENSP00000412197.2:p.Gln766Lys | ENST00000438665.2:c.2296C>A | -0.71 | neutral | 1.9x10-3 | 0.173 | benign | 40.4 | - | het in Fam2.II.1 and Fam2.I.1 | OMIM *606538 | GO BP | regulation of bicellular tight junction assembly (GO:2000810) |
| Fam2 | PKP2 | 3 | rs1800273 | X | 31986607 | G | A | missense | DMD | ENSP00000367948.2:p.Arg2151Trp | ENST00000378677.2:c.6451C>T | -1.37 | neutral | 0.026 | NA | NA | 25.1 | - | hemi in Fam2.II.1 and Fam2.I.1; het in Fam2.I.2 | Recessive mutations in DMD can cause muscle dystrophy (OMIM *300377). | GO BP | cardiac muscle cell action potential (GO:0086001) |
| Fam2 | PKP2 | 4 | rs137853909 | 20 | 10389422 | T | C | missense | MKKS | ENSP00000382008.2:p.Ile339Val | ENST00000399054.2:c.1015A>G | -0.52 | neutral | 4.3x10-3 | 0.95 | pathogenic | 14.4 | - | het in Fam2.I.2 and Fam2.II.2 | Recessive mutations in MKKS can cause Bardet-Biedl syndrome (OMIM *604896). | GO BP | heart development (GO:0007507) |
| Fam2 | PKP2 | 5 | rs61752484 | 1 | 120469147 | T | C | missense | NOTCH2 | ENSP00000256646.2:p.Asp1327Gly | ENST00000256646.2:c.3980A>G | -0.83 | neutral | 0.012 | 0.795 | pathogenic | 6.3 | - | het in Fam2.I.1 and Fam2.II.1 | This variant has been reported causal for Congenital heart disease as compound heterozygote with L2408H, which is absent in Fam2 [17]. | GO BP | negative regulation of cell proliferation (GO:0008285) |
| Fam2 | PKP2 | 6 | rs189277711 | 16 | 2158022 | G | A | missense | PKD1 | ENSP00000456672.1:p.Arg198Trp | ENST00000488185.2:c.591C>T | 0 | neutral | 6.9x10-3 | NA | NA | 7.1 | - | het in Fam2.II.1 and Fam2.I.1 | Dominant mutations have been associated with polycystic kidney disease (OMIM *601313). | GO BP | heart development (GO:0007507) |
| Fam2 | PKP2 | 7 | rs1805124 | 3 | 38645420 | T | C | missense | SCN5A | ENSP00000398962.2:p.His558Arg | ENST00000414099.2:c.1673A>G | 3.92 | neutral | 0.221 | 0.083 | benign | 22.2 | - | het in Fam2.I.1, Fam2.I.2, Fam2.II.2, Fam1.II.3 | This variant has been reported causal for isolated conduction disease as compound heterozygote with T215I, which is absent in Fam2 [18]. | PPI / GO BP | STRING score=903; positive regulation of sodium ion transport (GO:0010765); regulation of heart rate by cardiac conduction (GO:0086091) |
| Fam2 | PKP2 | 8 | rs536181176 | 17 | 12647691 | CCAG | C | inframe deletion | MYOCD | ENSP00000341835.4:p.Gln304del | ENST00000343344.4:c.910_912delCAG | 0.15 | neutral | 0.024 | NA | NA | 8 | - | het in Fam2.II.1 and Fam2.I.1 | Cardiac muscle-specific transcriptional coactivator of serum response factor. Mutations have been associated with hypertrophic cardiomypathy (OMIM *606127). | GO BP | negative regulation of cell proliferation (GO:0008285) |
| Fam2 | PKP2 | 9 | rs985861 | 18 | 28710619 | C | A | missense | DSC1 | ENSP00000257198.5:p.Cys848Phe | ENST00000257198.5:c.2543G>T | 0.56 | neutral | 0.05 | 0.039 | benign | 13.9 | - | het in Fam2.II.1 and Fam2.I.1 | Desmosomal protein desmocolin 1 (*OMIM 125643). | PPI | STRING score=901; MENTHA;SCORE=0.309 |
| Fam2 | PKP2 | 10 | rs55656741 | 5 | 31515657 | G | A | missense | DROSHA | ENSP00000339845.3:p.Ser321Leu | ENST00000344624.3:c.962C>T | -0.65 | neutral | 0.392 | 0.13 | benign | 6.7 | - | hom in Fam2.I.2, Fam2.II.2. Het in all others except Fam1.II.2 | Ribonuclease III. Mutations have been associated with cancer (OMIM *608828). | PPI | MENTHA score=0.126 |

**References**

1. Li H, Durbin R. Fast and accurate short read alignment with Burrows-Wheeler transform. Bioinformatics. 2009;25:1754-1760.

2. García-Alcalde F, Okonechnikov K, Carbonell J, et al. Qualimap: evaluating next-generation sequencing alignment data. Bioinformatics. 2012;28:2678-2679.

3. McKenna A, Hanna M, Banks E, et al. The genome analysis toolkit: A MapReduce framework for analyzing next-generation DNA sequencing data. Genome Res. 2010;20:1297-1303.

4. DePristo MA, Banks E, Poplin RE, et al. A framework for variation discovery and genotyping using next- generation DNA sequencing data. Nat Genet. 2011;43:491-498.

5. Auwera GA, Carneiro MO, Hartl C, et al. From FastQ data to high-confidence variant calls: the genome analysis toolkit best practices pipeline. Curr Protoc Bioinforma. 2013:10-11.

6. Jun G, Flickinger M, Hetrick KN, et al. Detecting and estimating contamination of human DNA samples in sequencing and array-based genotype data. Am J Hum Genet. 2012;91:839-848.

7. Ng PC, Henikoff S. Predicting Deleterious Amino Acid Substitutions Predicting Deleterious Amino Acid Substitutions. 2001:863-874.

8. Adzhubei IA, Schmidt S, Peshkin L, et al. A method and server for predicting damaging missense mutations. Nat Methods. 2010;7:248-249.

9. Cooper GM, Stone E a., Asimenos G, Green ED, Batzoglou S, Sidow A. Distribution and intensity of constraint in mammalian genomic sequence. Genome Res. 2005;15:901-913.

10. Weichenberger CX, Blankenburg H, Palermo A, et al. Dintor: functional annotation of genomic and proteomic data. BMC Genomics. 2015;16:1081.

11. Lek M, Karczewski KJ, Minikel E V., et al. Analysis of protein-coding genetic variation in 60,706 humans. Nature. 2016;536:285-291.

12. Choi Y, Sims GE, Murphy S, Miller JR, Chan AP. Predicting the Functional Effect of Amino Acid Substitutions and Indels. PLoS One. 2012;7:e46688.

13. König E, Rainer J, Domingues FS. Computational assessment of feature combinations for pathogenic variant prediction. Mol Genet Genomic Med. 2016;4:431-446.

14. Fromer M, Moran J, Chambert K, et al. Discovery and Statistical Genotyping of Copy-Number Variation from Whole-Exome Sequencing Depth. Am J Hum Genet. 2012;91:597-607.

15. Fromer M, Purcell SM. Using XHMM Software to Detect Copy Number Variation in Whole-Exome Sequencing Data. Curr Protoc Hum Genet. 2014:7-23.

16. Wessely R, Hengst L, Jaschke B, et al. A central role of interferon regulatory factor-1 for the limitation of neointimal hyperplasia. Hum Mol Genet. 2003;12:177-187.

17. Priest JR, Osoegawa K, Mohammed N, et al. De Novo and Rare Variants at Multiple Loci Support the Oligogenic Origins of Atrioventricular Septal Heart Defects. PLoS Genet. 2016;12:e1005963.

18. Viswanathan PC, Benson DW, Balser JR. A common SCN5A polymorphism modulates the biophysical effects of an SCN5A mutation. J Clin Invest. 2003;111:341-346.

19. Taylor M, Graw S, Sinagra G, et al. Genetic variation in titin in arrhythmogenic right ventricular cardiomyopathy-overlap syndromes. Circulation. 2011;124:876-885.

20. Morikawa Y, Heallen T, Leach J, Xiao Y, Martin JF. Dystrophin–glycoprotein complex sequesters Yap to inhibit cardiomyocyte proliferation. Nature. 2017;547: 227–231.

21. Cai Z, Wang Y, Yu W, Xiao J, Li Y, Liu L, et al. Hnulp1, a Basic Helix-Loop-Helix Protein With a Novel Transcriptional Repressive Domain, Inhibits Transcriptional Activity of Serum Response Factor. Biochem Biophys Res Commun. 2006;343:973–981.

22. Harrison SC, Cooper JA, Li K, Talmud PJ, Sofat R, Stephens JW, et al. Association of a sequence variant in DAB2IP with coronary heart disease. Eur Heart J. 2012;33:881–888.

23. Chinnadurai G. CtBP family proteins: More than transcriptional corepressors. BioEssays. 2003;25:9–12.

24 Covaceuszach S, Bozzi M, Bigotti MG, Sciandra F, Konarev PV, Brancaccio A, et al. Structural flexibility of human α-dystroglycan. FEBS Open Bio. 2017;7:1064–1077.
